# Altered glucuronidation deregulates androgen dependent response profiles and signifies castration resistance in prostate cancer

**DOI:** 10.18632/oncotarget.28059

**Published:** 2021-09-14

**Authors:** Brenna M. Zimmer, Michelle E. Howell, Linlin Ma, Jeffrey R. Enders, Danielle Lehman, Eva Corey, Joseph J. Barycki, Melanie A. Simpson

**Affiliations:** ^1^Department of Molecular and Structural Biochemistry, North Carolina State University, Raleigh, NC, USA; ^2^Department of Biochemistry, University of Nebraska, Lincoln, NE, USA; ^3^Molecular Education, Technology and Research Innovation Center, North Carolina State University, Raleigh, NC, USA; ^4^Department of Urology, University of Washington, Seattle, WA, USA; ^5^Lineberger Comprehensive Cancer Center, The University of North Carolina at Chapel Hill, Chapel Hill, NC, USA

**Keywords:** prostate cancer, castration resistance, detoxification, UDP-glucose dehydrogenase, patient-derived xenografts

## Abstract

Glucuronidation controls androgen levels in the prostate and the dysregulation of enzymes in this pathway is associated with castration resistant prostate cancer. UDP-glucose dehydrogenase (UGDH) produces UDP-glucuronate, the essential precursor for glucuronidation, and its expression is elevated in prostate cancer. We compared protein and metabolite levels relevant to the glucuronidation pathway in five prostate cancer patient-derived xenograft models paired with their isogenic counterparts that were selected *in vivo* for castration resistant (CR) recurrence. All pairs showed changes in UGDH and associated enzymes and metabolites that were consistent with those we found in an isogenic androgen dependent (AD) and CR LNCaP prostate cancer model. Ectopic overexpression of UGDH in LNCaP AD cells blunted androgen-dependent gene expression, increased proteoglycan synthesis, significantly increased cell growth compared to controls, and eliminated dose responsive growth suppression with enzalutamide treatment. In contrast, the knockdown of UGDH diminished proteoglycans, suppressed androgen dependent growth irrespective of androgens, and restored androgen sensitivity in CR cells. Importantly, the knockdown of UGDH in both LNCaP AD and CR cells dramatically sensitized these cells to enzalutamide. These results support a role for UGDH in androgen responsiveness and a target for therapeutic strategies in advanced prostate cancer.

## INTRODUCTION

Prostate cancer is the most frequently diagnosed cancer in US men, and the second most common cause of male cancer death [1]. Castration resistant (CR) recurrence following androgen deprivation therapy is a major cause of mortality in men with advanced prostate cancer. Tumors that recur after this treatment are highly aggressive and able to expand in conditions of low circulating androgens by one of several mechanisms, including aberrancies in androgen receptor (AR) expression or signaling, and upregulation of enzymes that control intratumoral androgen synthesis [2–8]. However, prostate epithelial cells also control the potency and availability of androgen hormones by inactivating and exporting them [9, 10]. There is still an incomplete understanding of how this mechanism may contribute to loss of androgen sensitivity in tumor recurrence.

Androgen inactivation by glucuronidation has been implicated in maintenance of androgen dependence in prostate tumor cells [11]. The metabolic precursor for glucuronidation, UDP-glucuronate, has three downstream fates in the prostate and the mechanism for prioritization among them is unknown. UDP-glucose dehydrogenase (UGDH) is a unique, essential enzyme with the pivotal role of providing UDP-glucuronate (UDP-GlcA) for use by UDP-glucuronosyltransferase (UGT) enzymes in glucuronidation [12]. UGDH activity is also required to support biosynthetic processes: UDP-GlcA initiates specifically timed production of proteoglycans in the Golgi and is required to produce hyaluronan at the plasma membrane. Thus, although UGDH is a cytosolic enzyme, its product is needed for compartmentally segregated, energy-competitive processes, and the pools of UDP-GlcA must be tightly regulated.

Several recent reports have implicated UGDH as an essential promoter of cell migration, invasion, and metastasis in lung and breast cancer models [13–16]. Our previous studies in prostate cancer models found that UGDH levels are androgen stimulated and increased UGDH can drive intracellular steroid depletion through elimination as androgen-glucuronides [17]. This mechanism is significantly more robust in prostate tumor cells that remain dependent on exogenous androgen. The inhibition or loss of androgen-stimulated UGDH impacts androgen-glucuronide secretion, UGT expression, and proliferation rate of androgen dependent cells [17]. This result provides key evidence that UGDH activity is a limiting factor in the release of androgen from tumor cells. In contrast, UGDH is elevated in CR prostate cancer and the increase in those cells is associated with higher hyaluronan production and proteoglycan expression [18]. Despite also having higher glucuronidation potential, glucuronide output is significantly reduced, likely indicating an intracellular prioritization scheme that is linked to precursor availability.

In the present study, our goals were to examine the gene expression and UDP-sugar profile changes in human prostate cancer patient-derived xenograft (PDX) pairs representing castration sensitive (CS) and CR tumors, each pair originally from the same patient, and to determine whether UGDH served as a control point for directed channeling of UDP-GlcA through its downstream fates as reflected in tumors. To address the impact of manipulating UGDH levels on enzyme expression and metabolic outputs, we used an isogenic cell culture model of androgen dependent (AD) and CR prostate cancer, LNCaP 33 and 81, respectively, as previously described [17, 18]. Using this model, we compared the impact of UGDH manipulation on AR-mediated gene expression, proteoglycan production, glucuronidation enzymes, UDP-sugar metabolites, and the proliferation rates of tumor cells. Collectively, our results support a model in which UGDH expression levels can selectively control the androgen elimination pathway in prostate tumor cells, where excess UGDH drives castration resistance while reduction of UGDH may permit retention or re-establishment of androgen sensitivity.

## RESULTS

### Patient-derived xenograft (PDX) models of CRPC reflect alteration in nucleotide sugar precursors and the components of their utilization pathways that are reprogrammed in UGDH-manipulated tumors

We previously reported a detailed examination of gene expression and metabolite levels in pathways impinging on the enzyme UGDH in the LNCaP model of androgen dependent (AD, also termed castration sensitive) and castration resistant (CR) prostate cancer. UGDH produces the multifunctional precursor UDP-GlcA, which is critical for levels of intracellular androgen as well as biosynthetic products such as the proteoglycan Notch1. The prior analysis revealed a characteristic association between levels of specific enzymes and metabolites (summarized in Table 1), and the loss of androgen response [18].

**Table 1:** Summary of components and reason for analysis

UGDH	Converts UDP-Glc to UDP-GlcA
UDP-Glc	Substrate for UGDH
UDP-GlcA	Product of UGDH
UDP-Xyl	Metabolite of UDP-GlcA required for proteoglycan initiation
UGT2B17	AR-suppressed gene
FoxA1	Required for transcription of UGT2B17
UXS1	Converts UDP-GlcA to UDP-Xyl
FL Notch1	Proteoglycan requiring UDP-Xyl for correct cell surface expression
NTM Notch1	N-terminal transmembrane portion of Notch1 indicating surface processing
AR	Mediates androgen transcriptional response
PSA	AR-stimulated gene

**Figure 1:**
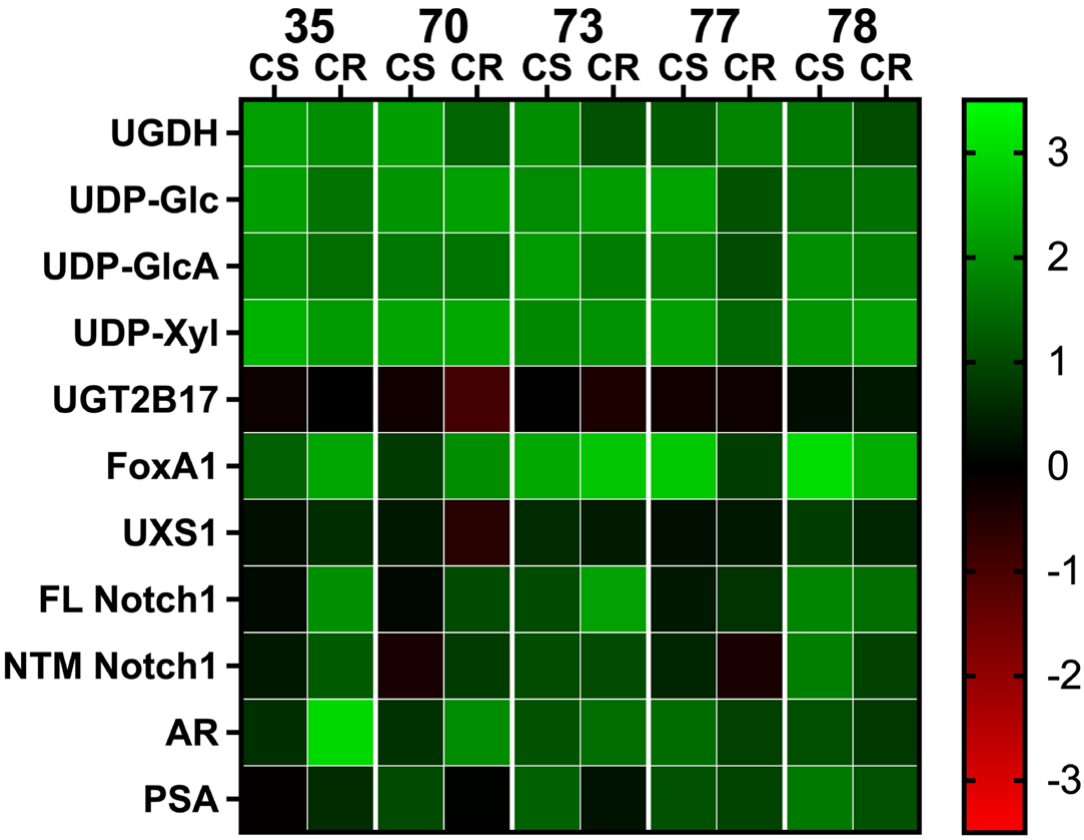
Comparative analysis of gene expression and metabolite levels in PDX tissue. Changes in gene expression and metabolite levels among the LuCaP PDX models are plotted in a heat map using a double log scale. Colors indicate changes in gene expression or metabolite levels relative to LNCaP tumors grown in mice. On the scale, green denotes an increase in protein or metabolite; red denotes a decrease. Abbreviation: CS: castration sensitive; CR: castration resistant. Numbers indicate respective PDX models summarized in Table 2.

To validate and expand these associations in clinical specimens, we analyzed five paired CS/CR isogenic PDX models from the LuCaP series [19]. The LuCaP PDX collection is a clinically well characterized cohort of human prostate tumors developed in a consistent manner from resected primary tumors and/or metastases recovered through a rapid autopsy program. Clinical characteristics of the models and selected information from each patient history are summarized in Table 2. Of the five PDX models analyzed, LuCaP 73 was derived from a primary tumor, and the others are from metastases. In addition, multiple PDX tumors in this collection have been used successfully to derive isogenic CR tumors in castrated mice. Extensive molecular and cellular analysis of these models confirmed that they accurately represent and retain the considerable heterogeneity of human prostate cancer, and the characteristics of the original tumor [19, 20]. Thus, they offer excellent models in which to examine the underlying mechanisms of CR recurrence. We first measured the gene expression and metabolite components that comprise the glucuronidation and proteoglycan profiles we identified previously in cell lines (Table 1). The status of the profile components was compiled and presented as a heat map, in which changes within the PDX models are plotted on a logarithmic scale relative to the profile we measured in LNCaP tumors grown in SCID mice (Figure 1). Not surprisingly, there was considerable individual variability. However, as noted in the published characterization of the PDX tissues, AR and FoxA1 were significantly elevated in all five CS PDX and increased further in 4/5 of the isogenic CR PDX pairs. Despite increases in the AR and FoxA1 transcriptional regulators, expression of target proteins PSA and UGT2B17 was not consistently associated. There were several additional noteworthy differences between the CS PDX tumors and LNCaP tumors, including significant changes in the components of the glucuronidation associated profile. In particular, UGDH and the UDP-sugars that are directly used and affected by UGDH were all elevated multi-fold, and UGT2B17 expression was moderately lower. Proteoglycan level as reflected by Notch1, which requires UDP-GlcA and UDP-Xyl for its correct expression, was also significantly elevated in all five CS PDX tissues.

**Table 2:** Description of PDX characteristics (adapted from [19])

PDX	35		70		73		77		78	
Tissue	Lymph node		Liver		Prostate		Femur		Lymph node	
Source^a^	OR		TAN		OR		TAN		TAN	
Gleason	5 + 5		3 + 4		4 + 5		not available		7	
Oncogenes^b^	NKX3.1 loss of heterozygosity, MYC copy number gain									
Hormone treatments	All patients had CRPC following androgen deprivation therapy, and subsequently received secondary diethylstilbestrol									
Additional treatment^c^	None		Cort		Keto, cort		Mitoxantrone		Keto, cort, taxol/taxotere	
AR status	Increased		Increased		Increased, mutated		Increased		Increased, mutated	
**PDX**	**35**	**35R**	**70**	**70R**	**73**	**73R**	**77**	**77R**	**78**	**78R**
PSA status^d^	Lo	Lo	Med	Lo	Med	Lo	Hi	Med	Med	Med
DHT	nc	nc	nc	nc	nc	High	nc	nc	nc	High

^a^OR: operating room during resection; TAN: tissue acquisition autopsy. ^b^Representative, not a comprehensive list. ^c^Cort, corticosteroids, palliative to combat side effects of ADT; Keto, ketoconazole, CYP17A1 inhibitor; Mitoxantrone, cytotoxic chemotherapeutic. ^d^PSA status Lo: 5–100 nM; Med: 99–500 nM; Hi: >500 nM.

A comparison of protein and metabolite levels between the CS and CR paired PDX models showed that many of the changes to UGDH and the associated partitioning enzymes and metabolites were congruent to those we reported in the LNCaP AD and CR cell lines (Figure 1 and numeric data summarized in Supplementary Table 1). Although there was variability among PDXs as expected given the heterogenous patient background and tissue of origin, it was clear that all pairs reflected changes in glucuronidation pathway profiles that correlated with the transition from CS to CR. UGT2B17 expression was generally slightly lower in CS PDX than in the LNCaP tumors, but levels decreased slightly further in CR PDX despite the tumors having been exposed to lower circulating androgen in the castrated state. UGDH levels decreased in 4/5 CR PDX relative to the CS counterpart, which would be expected from reduced androgen availability, but importantly, the expression of UGDH was still considerably higher than in the LNCaP tumors. Consistent with dramatically elevated UGDH expression, UDP-sugars were also present at high concentrations, and the level of Notch1 proteoglycan increased in all five CR PDX relative to CS counterparts. Collectively, the pattern of altered expression and metabolite levels is consistent with probable functional alterations to UGDH and/or one or more of the enzymes downstream of the UDP-GlcA product, resulting in increased proteoglycan production at the cost of androgen glucuronidation.

### Overexpression of UGDH in the AD LNCaP background desensitizes AR-mediated gene expression and reduces glucuronidation precursors

**Figure 2:**
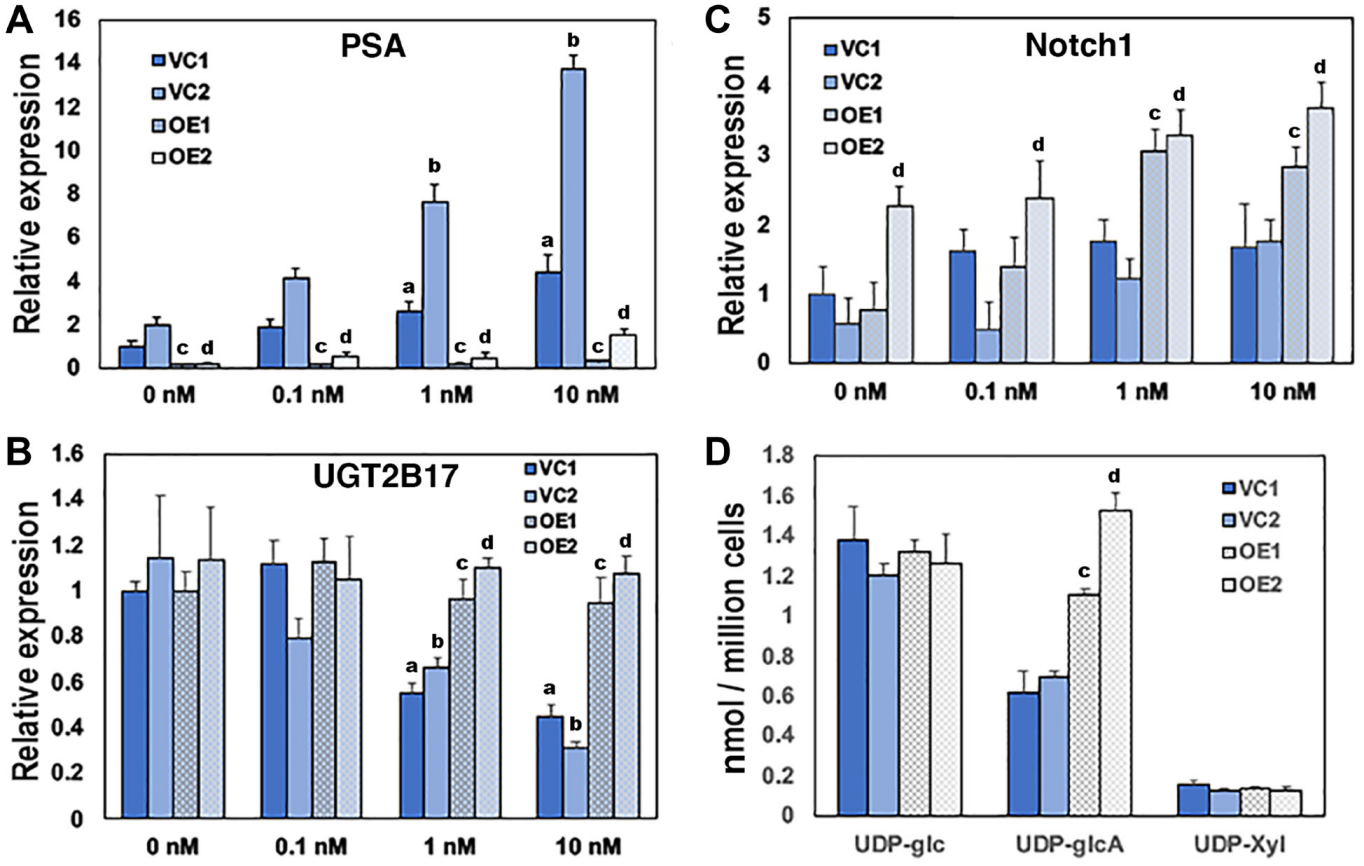
Overexpression of UGDH in the AD LNCaP background desensitizes AR-mediated gene expression and reduces glucuronidation precursors. Two vector control cell lines (VC1 and VC2) and two UGDH-overexpressing lines (OE1 and OE2) were selected in the LNCaP AD background. Equal cell counts were seeded 48 hours in androgen depleted media, followed by removal and replacement with media containing DMSO (vehicle, 0 nM) or the indicated concentration of DHT. After an additional 48 hours, cells were harvested for analysis. AR-dependent genes PSA (**A**) and UGT2B17 (**B**) were analyzed by WB in whole cell lysates; (**C**) functional synthetic output of each cell line was assessed by Notch1 expression in cell lysates; (**D**) UDP-sugar pools were measured in cell lysates by LC-MS. In panels A–C, mean ± SEM is plotted for triplicate measurements. In panel D, mean ± SD is plotted for quadruplicate measurements. Statistical significance is indicated as: (a) *p* < 0.05 relative to VC1 at 0 nM DHT. (b) *p* < 0.05 relative to VC2 at 0 nM DHT. (c) *p* < 0.05 comparing OE1 to both VC1 and VC2 at the indicated [DHT]. (d) *p* < 0.05 comparing OE2 to both VC1 and VC2 at the indicated [DHT].

To determine whether UGDH was responsible for the associated metabolic alterations observed in the transition of cell lines and PDX tumors from CS to CR, we overexpressed UGDH in the LNCaP model and examined the impact of increased UGDH levels on androgen dependent gene expression and nucleotide sugars. LNCaP AD cells (ATCC) are androgen dependent and AR positive. LNCaP CR cells were derived from LNCaP AD in cultures selected for loss of androgen sensitivity [21]. LNCaP CR cells intrinsically express higher levels of UGDH and UGT2B17 than the LNCaP AD cells and the magnitude of AR-mediated gene expression is blunted several-fold in LNCaP CR cells [18]. Four clones were selected and characterized in each background. Two lines overexpressed UGDH (OE1 and OE2) and two control lines contained the vector only (VC1 and VC2, Supplementary Figures 1A and 2A). AR expression was not consistently altered in the absence or presence of DHT in LNCaP AD cells (Supplementary Figure 1B). However, both VC clones exhibited DHT-dependent increases in PSA expression (Figure 2A) and UGT2B17 suppression (Figure 2B), as characteristic of the LNCaP AD parents. In contrast, OE1 and OE2 lines expressed almost no PSA and it was minimally stimulated by DHT at any dose (Figure 2A). UGT2B17 expression was similar in all lines in the absence of DHT, but unlike the VC clones, DHT addition did not suppress UGT2B17 in UGDH OE lines (Figure 2B). Relative gene expression and metabolite levels in all LNCaP UGDH VC and OE cells are summarized in Supplementary Table 2 and presented as heat maps in Supplementary Figure 6A and 6B.

Analysis of UDP-sugar pools and flux through pathways downstream of UDP-GlcA production has shown that in CR cells, more UDP-GlcA is sent to proteoglycan and glycosaminoglycan biosynthesis, increasing the abundance of tumor-promoting cell surface and extracellular matrix components [18]. Western analysis of both full length and processed Notch1 proteoglycan revealed that both were significantly elevated in UGDH-overexpressing cells (Figure 2C). As expected, UDP-GlcA pools were increased significantly in UGDH OE lines (Figure 2D). The comparatively low UDP-Xyl levels remained constant in the UGDH OE lines, consistent with basally higher use for the initiation of glycan chains on Notch1 core proteins and reflecting the increased direction of UDP-GlcA to proteoglycan synthesis.

### Overexpression of UGDH in the CR LNCaP background further suppresses AR-mediated expression of glucuronidation genes and reduces nucleotide sugar pools without impacting proteoglycan production

**Figure 3:**
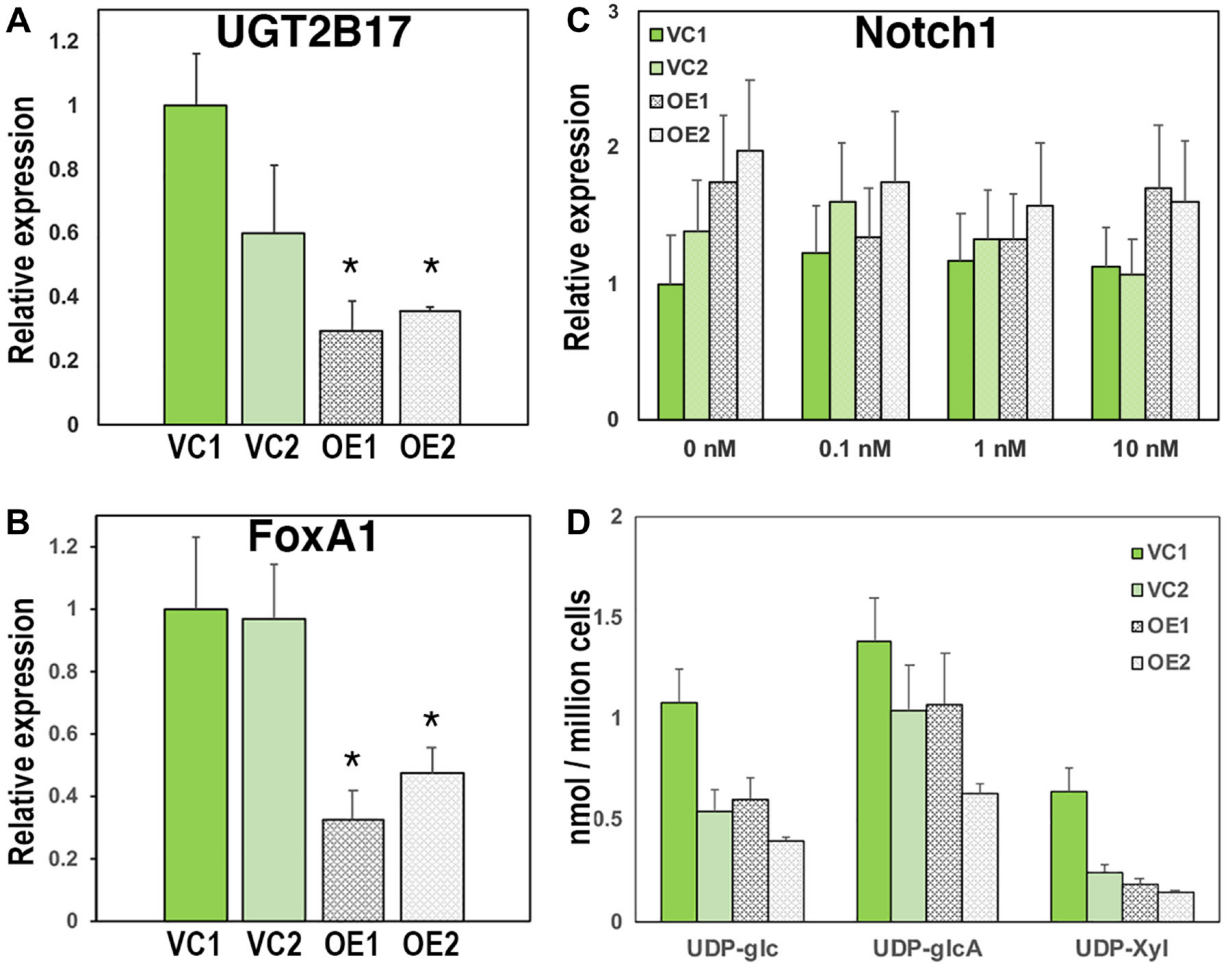
Overexpression of UGDH in the CR LNCaP background further suppresses AR-mediated expression of glucuronidation genes and reduces nucleotide sugar pools without impacting proteoglycan production. Two vector control cell lines (VC1 and VC2) and two UGDH-overexpressing lines (OE1 and OE2) were selected in the LNCaP CR background. Equal cell numbers were seeded 48 hours in androgen replete media followed by harvest of cells for analysis of gene expression (**A** and **B**) and UDP-sugars (**D**). For panel (**C**), equal cell counts were seeded 48 hours in androgen depleted media, followed by removal and replacement with media containing DMSO (vehicle, 0 nM) or the indicated concentration of DHT. After an additional 48 hours, cells and media were harvested for analysis. AR-dependent genes UGT2B17 (A) and FoxA1 (B) were analyzed by WB in whole cell lysates; (C) functional synthetic output of each cell line was assessed by Notch1 expression in cell lysates; (D) UDP-sugar pools were measured in cell lysates by mass spectrometry. Mean ± SEM is plotted for triplicate technical measurements; ^*^ *p* < 0.05 for OE1 and OE2 relative to VC1 and VC2.

When UGDH overexpression was characterized in LNCaP CR cells (Supplementary Figure 2A), PSA expression was low and remained unchanged, as seen in the parent line (Supplementary Figure 2B). AR expression was unaffected by UGDH OE and was not significantly altered by DHT (Supplementary Figure 2C). The expression of UGT2B17 was significantly reduced by UGDH OE relative to the vector controls (≈50–75%), but the expression was not suppressed by DHT addition (Figure 3A and Supplementary Figure 3A). FoxA1 was found to be lower by 60–70%, commensurate with the reduced expression of its transcriptional target UGT2B17 (Figure 3B and Supplementary Figure 3B). Notch1 and UXS1 expression were unchanged and not androgen responsive (Figure 3C and data not shown). UDP-sugar levels were somewhat variable among cell lines (Figure 3D), but the ratios of product to substrate among lines remained constant, indicating comparable steady state flux through those enzymes and consistent with unchanged Notch1 expression. Therefore, UGDH overexpression in LNCaP cells that were already androgen insensitive had limited additional impact on biosynthetic output, but further disrupted the expression profiles of androgen transcriptional regulators and elimination enzymes.

### UGDH overexpression in both AD and CR LNCaP cells promotes androgen independent growth

**Figure 4:**
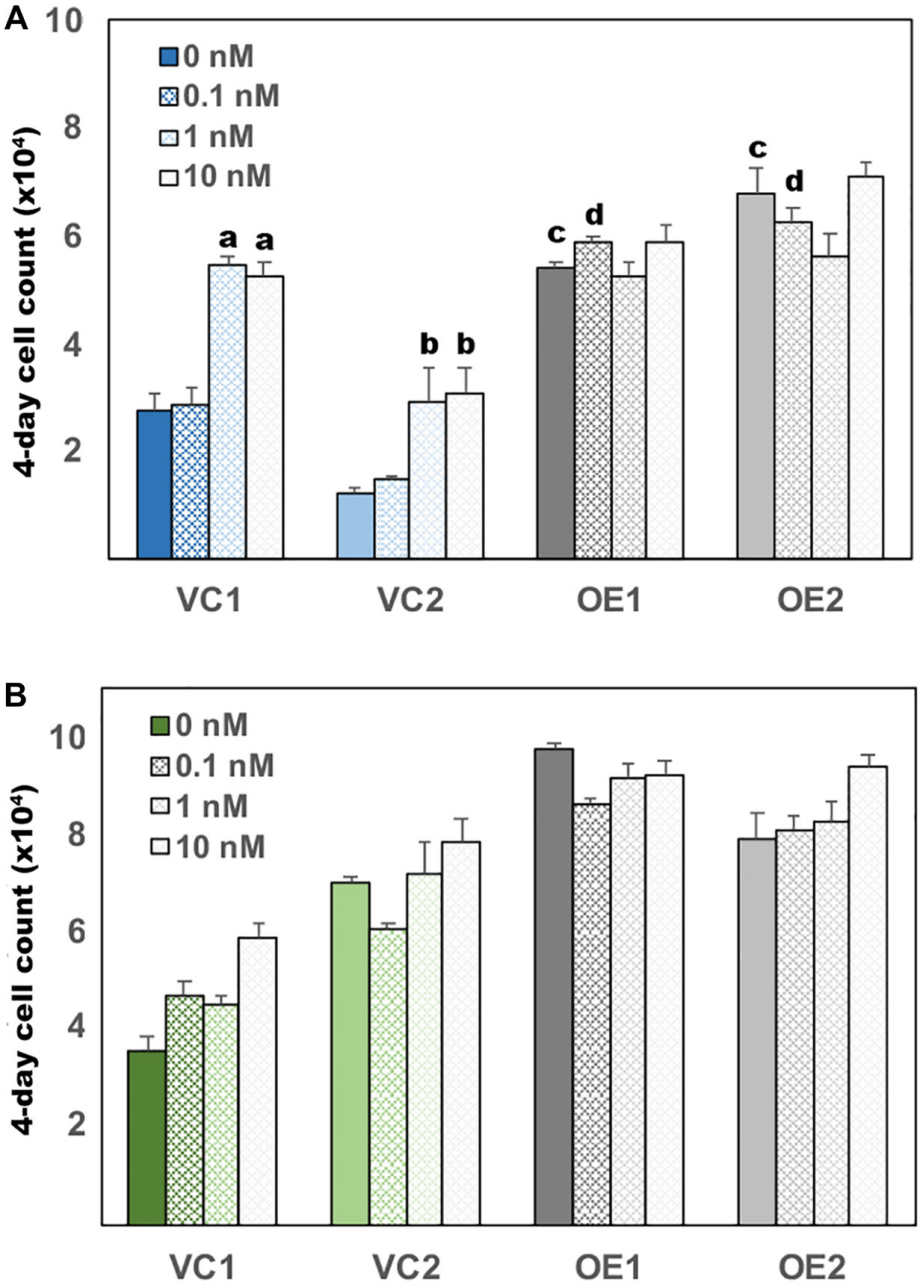
UGDH overexpression in both AD and CR LNCaP cells promotes androgen independent growth. Equal numbers of the indicated transfectants in the AD (**A**) or CR (**B**) LNCaP cell model were seeded 48 hours in androgen depleted media, followed by removal and replacement with media containing DHT or DMSO (vehicle, 0 nM). Each day, cell numbers were assessed in quadruplicate wells for each line by addition of resazurin and comparison of the resulting resorufin fluorescence relative to a standard curve. Four-day cell counts for each line were normalized to the respective counts on day one. Mean fold increase in the 4-day assay ± SEM is plotted. Statistical significance is indicated as: (**a**) *p* < 0.05 relative to VC1, 0 nM DHT. (**b**) *p* < 0.05 relative to VC2, 0 nM DHT. (**c**) *p* < 0.05 relative to VC1 and VC2 at 0 nM DHT. (**d**) *p* < 0.05 relative to VC1 and VC2 at 0.1 nM DHT.

LNCaP AD cells proliferate poorly in androgen-depleted conditions and respond in a concentration-dependent manner to the exogenous addition of DHT, while LNCaP CR cells rapidly proliferate even in the absence of androgen [17, 18]. To test whether altered metabolite use upon UGDH overexpression promoted androgen independent growth, we next compared proliferation rates of the subclones in the presence and absence of DHT. LNCaP AD cells expressing the vector control exhibited the expected lag in proliferation upon complete androgen withdrawal, and grew only upon addition of 1 nM or 10 nM DHT, as previously observed in the parent line (Figure 4A). In contrast, UGDH overexpression in LNCaP AD cells was accompanied by a rapid increase in growth in androgen-free vehicle-treated conditions (0 nM, Figure 4A), attaining two-fold higher cell numbers at 0 nM DHT than VC1 or VC2 LNCaP AD cells at 10 nM DHT. Growth of the UGDH OE subclones in LNCaP AD cells was no longer androgen responsive, and cells reached comparable density at all doses of DHT (Figure 4A). LNCaP CR cells intrinsically grow more rapidly in 10 nM DHT than the LNCaP AD cells. As expected, LNCaP CR cells expressing the vector control grew in the absence of DHT and growth was only modestly stimulated by 10 nM DHT (Figure 4B). Also as expected, androgen dependent proliferation was not affected by UGDH overexpression. However, UGDH OE cells did grow faster in all conditions by at least 20% relative to VC cells (Figure 4B). Together, these results support a role for elevated UGDH in driving androgen independent tumor cell growth through altered use of UDP-sugar metabolites.

### Loss of UGDH promotes AR-dependent gene expression and reduces proteoglycan production while sustaining UDP-sugar flux

**Figure 5:**
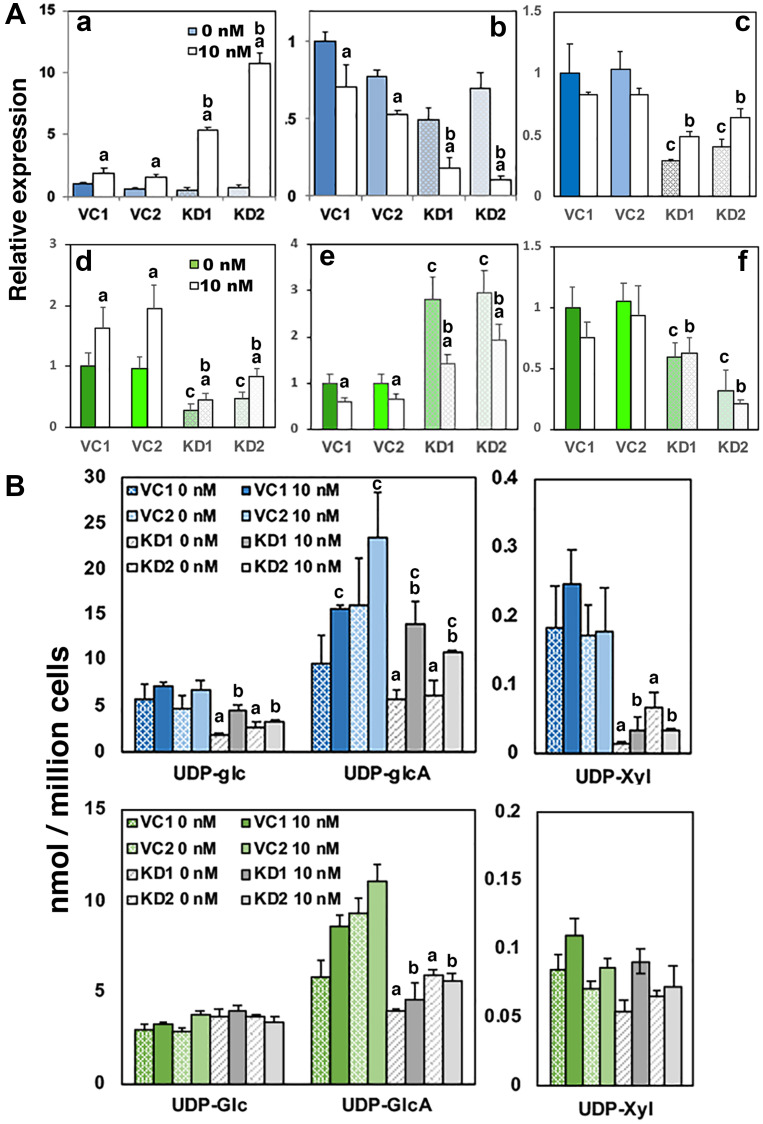
Loss of UGDH promotes AR-dependent gene expression and reduces proteoglycan production while sustaining glucuronide output. LNCaP AD and CR cells were selected for stable expression of a non-targeting vector control (VC1 and VC2) or a UGDH shRNA knockdown construct (KD1 and KD2). Equal cell counts were seeded 48 hours in androgen depleted media, followed by removal and replacement with media containing 10 nM DHT or DMSO (vehicle, 0 nM). After an additional 48 hours, cells were harvested for analysis by western blot and mass spectrometry. (**A**) AR-dependent genes PSA (panels **a** and **d**) and UGT2B17 (panels **b** and **e**) were analyzed by WB in whole cell lysates; functional synthetic output of each cell line was assessed by Notch1 expression in cell lysates (panels **c** and **f**). Panels (a–c) depict expression data in the AD background and panels (d–f) illustrate data from the CR background. Mean ± SEM is plotted. Statistical significance is indicated on the plots as: (a) *p* < 0.05 for that clone, comparing 0 vs 10 nM DHT; (b) *p* < 0.05 relative to VC1 and VC2, 10 nM DHT; (c) *p* < 0.05 relative to VC1 and VC2, 0 nM DHT. (**B**) UDP-sugar pools were measured in cell lysates by LC-MS for both the AD (upper) and CR (lower) backgrounds as indicated. Mean ± SD is plotted. Statistical significance is indicated as: (a) *p* < 0.05 for both KD1 and KD2 relative to VC1 or VC2, 0 nM DHT. (b) *p* < 0.05 for both KD1 and KD2 relative to VC1 or VC2, 10 nM DHT. (c) *p* < 0.05 for that clone, comparing 0 and 10 nM DHT.

To test the requirement for UGDH in androgen dependent parameters, we next compared androgen-regulated gene expression and UDP-sugar pools in LNCaP AD and CR cells clonally selected for shRNA knockdown of UGDH. Androgen deprivation for 48 hours reduced UGDH levels in both control and UGDH KD cells (Supplementary Figure 4A and 4B). UGDH protein expression, as expected, was significantly stimulated only in LNCaP AD subclones, and moderately increased in both control and UGDH KD cells upon addition of DHT (Supplementary Figure 4A and 4B). AR expression was similar between UGDH KD and VC cells and did not vary with DHT (Supplementary Figure 5A and 5B). In LNCaP AD, PSA was significantly higher in UGDH KD cells in standard media relative to controls (not shown), but expression dropped to the same low levels in androgen free media (Figure 5A, panel a). DHT stimulation resulted in ≈3-fold increases in the expression of PSA in VC cells, but the magnitude of the increase in the UGDH KD cells was ≈5-fold and ≈11-fold for KD1 and KD2, respectively. Similarly and as expected, UGT2B17 expression was elevated 2–3 fold in the absence of androgen (data not shown), and was suppressed by ≈30% in the control cells with addition of DHT (Figure 5A, panel b). Strikingly, when DHT was added to UGDH KD1 and KD2 lines, UGT2B17 expression was reduced by ≈65% and 90%, respectively. Notch1 expression was significantly diminished (by 60–70%) upon knockdown of UGDH, but was not altered by DHT either in VC or KD cells (Figure 5A, panel c).

When UGDH was knocked down in LNCaP CR cells, which already express relatively little PSA, the level of PSA expression was further reduced (≈60% of non-targeting controls in the absence of DHT, Figure 5A, panel d). Although there was residual DHT stimulation, PSA levels remained ≈30% of the control level. Similarly, UGDH KD strongly derepressed UGT2B17 upon androgen depletion, and partially restored the androgen suppressive effect (≈35–50% comparing absence and presence of DHT, Figure 5A, panel e). Notch1 proteoglycan expression at the cell surface was diminished by UGDH knockdown irrespective of androgen, as we previously observed in standard media (Figure 5A, panel f). Relative gene expression and metabolite levels in all LNCaP UGDH VC and KD cells are summarized in Supplementary Table 2 and presented as heat maps in Supplementary Figure 6C and 6D.

Comparison of the UDP sugar pools in LNCaP AD control and UGDH KD cells revealed significant decreases in UDP-GlcA in UGDH KD cells that could be stimulated by ≈2-fold with DHT addition. UDP-Xyl levels were 80–90% lower in UGDH KD cells (Figure 5B, upper panels). Cellular ratios of the product UDP-GlcA to substrate UDP-Glc were similar in both control and UGDH KD cells. However, the significant reduction in UDP-Xyl resulting from diminished UDP-GlcA availability, is consistent with the loss of Notch 1 expression in these cells. Importantly, the knockdown of UGDH in the LNCaP CR background also resulted in significant reduction of UDP-GlcA in both absence and presence of DHT (Figure 5B, lower panels). In this case, levels of UDP-Xyl were already much lower, on the order of those measured in the LNCaP AD lines with UGDH knocked down, and were not further reduced. These results show that diminishing UDP-GlcA through loss of UGDH expression leads to reduction of cell surface proteoglycans such as Notch 1. Considering the collective effects on UDP-GlcA, proteoglycans, and the increased sensitivity of androgen-mediated gene expression above, the implication is that there is reprioritization of this multifunctional precursor from proteoglycan synthesis to androgen glucuronidation based on levels of UGDH activity.

### Loss of UGDH eliminates growth of AD LNCaP cells and restores partial androgen sensitivity to CR cells

**Figure 6:**
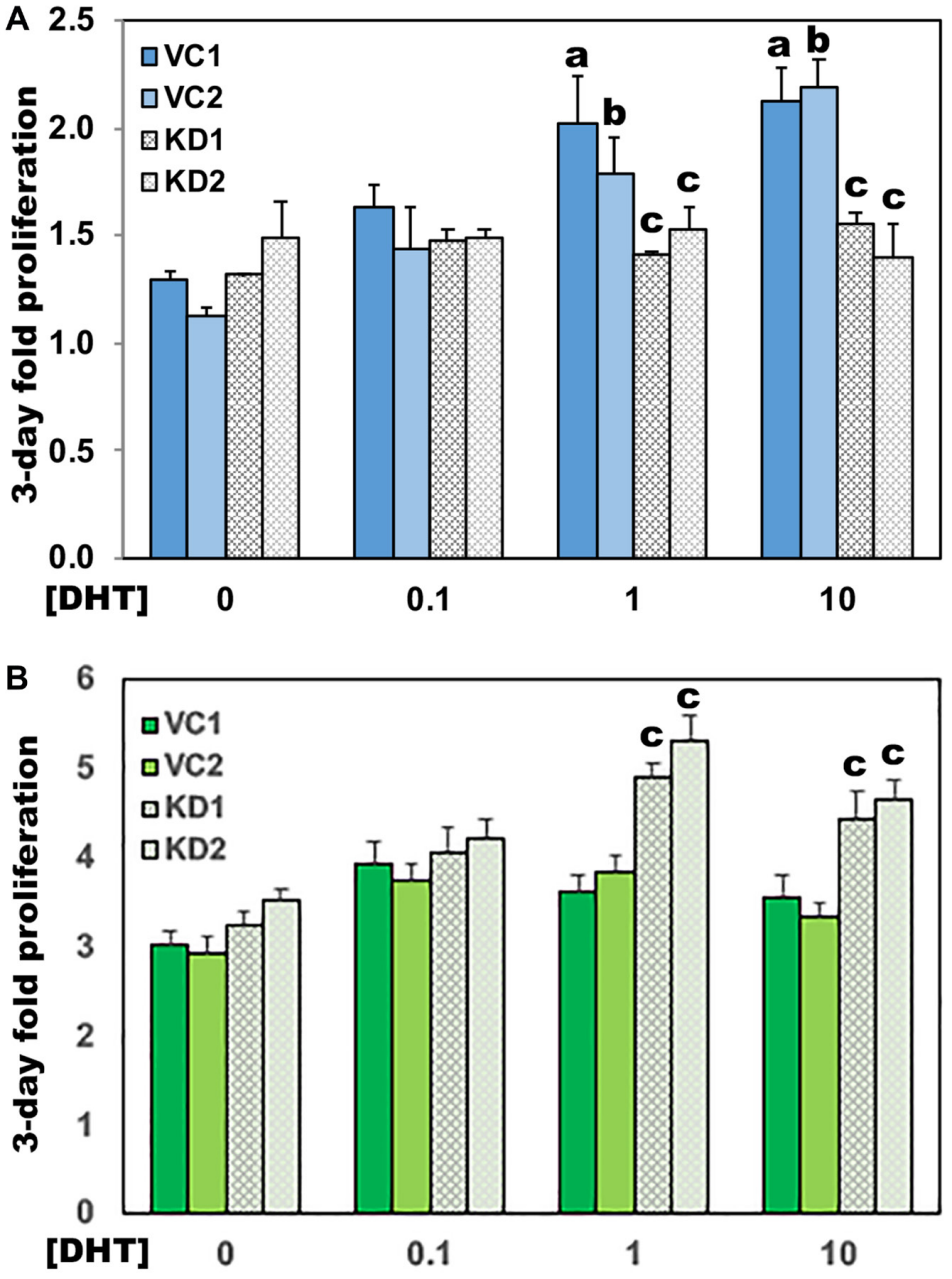
Loss of UGDH eliminates growth of AD LNCaP cells and restores partial androgen sensitivity to CR cells. Equal numbers of the indicated transfectants in the AD (**A**) or CR (**B**) LNCaP cell model were seeded 48 hours in androgen depleted media, followed by removal and replacement with media containing DHT or DMSO (vehicle, 0 nM). Each day, cell numbers were assessed by addition of resazurin and comparison of the resulting resorufin fluorescence relative to a standard curve. Fold proliferation is plotted as in Figure 4, and represents the mean of four technical replicates ± SEM. Statistical significance is indicated as: (**a**) *p* < 0.05 relative to VC1, 0 nM DHT. (**b**) *p* < 0.05 relative to VC2, 0 nM DHT. (**c**) *p* < 0.05 relative to VC1 and VC2 at the indicated [DHT].

Availability of androgens within the prostate tumor cell has been shown to promote growth and survival of cells in androgen depleted conditions [17]. We therefore tested the effect of UGDH knockdown on androgen-dependent proliferation. While VC cell density significantly increased at both 1 nM and 10 nM DHT concentrations (Figure 6A), the UGDH KD cells failed to thrive at any dose, and began to exhibit reduced density by day four. Thus, UGDH KD impaired androgen dependent cell growth regardless of exogenous DHT concentrations or intracellular synthesis, likely as a result of increased glucuronidation potential. In contrast, the knockdown of UGDH in LNCaP CR cells did not inhibit cell growth (Figure 6B). However, UGDH knockdown had the effect of increasing sensitivity to exogenous androgen, since the LNCaP CR UGDH KD cells had partially restored androgen responsiveness at 1 nM and 10 nM DHT relative to VC cells.

### UGDH manipulation regulates concentration dependence of enzalutamide growth suppression

**Figure 7:**
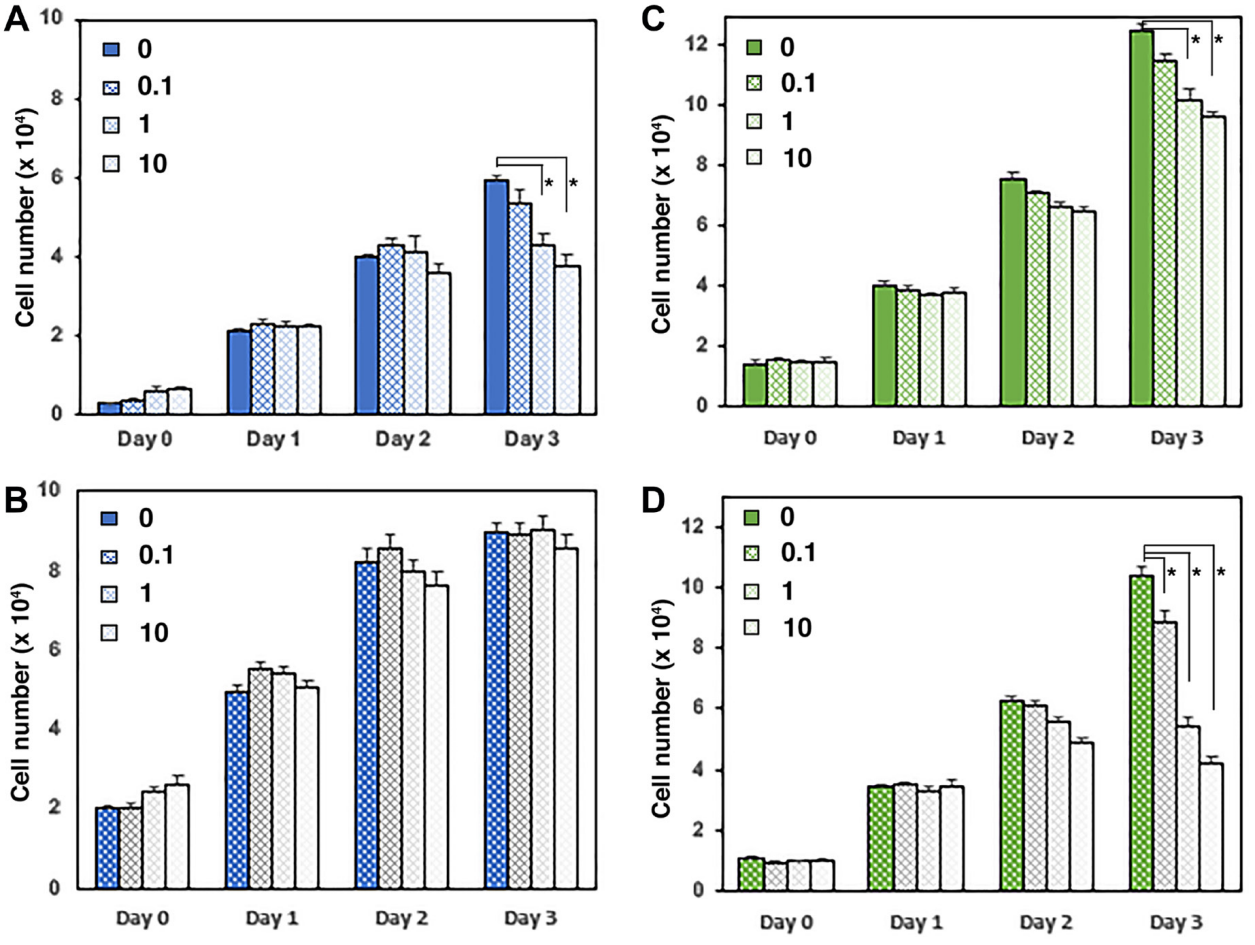
UGDH manipulation regulates concentration dependence of enzalutamide growth suppression. (**A** and **B**) LNCaP AD cells overexpressing UGDH (B) were compared to vector control (A) and to LNCaP CR cells with UGDH knocked down (**C**, Control; **D**, UGDH KD). Equal cell numbers were plated and treated for three days with the indicated concentrations of enzalutamide (in mM). Proliferating cells were quantified in a fluorescence plate reader using resazurin-resorufin conversion. Daily cell count was determined from a standard curve and represents the mean of four technical replicates ± SEM; ^*^ *p* < 0.05.

Given the dramatic effect of UGDH knockdown on androgen sensitive cell growth, we tested the response of the UGDH manipulated cells to the anti-androgen enzalutamide, which normally suppresses growth of LNCaP AD cells. As expected, 1 μM and 10 μM concentrations of enzalutamide reduced the density of LNCaP AD control cells by ≈45% at 3 days relative to the vehicle treated condition (Figure 7A). In contrast, the overexpression of UGDH in LNCaP AD cells increased their intrinsic growth rate by ≈25%, and eliminated the dose responsive growth suppression observed with enzalutamide treatment (Figure 7B). LNCaP CR cells exhibited little sensitivity to enzalutamide: as expected, the VC cells showed ≈10–20% reduced growth in 1 μM and 10 μM conditions (Figure 7C). In contrast, the knockdown of UGDH in LNCaP CR cells reduced their intrinsic growth rate by 20% and dramatically sensitized these cells to enzalutamide, reducing 3-day growth by >65% with 10 μM treatment relative to the vehicle (Figure 7D). These results further support a role for UGDH in regulation of androgen responsiveness and highlight its potential as a target for therapeutic strategies in advanced prostate cancer.

## DISCUSSION

Androgen inactivation by glucuronidation is a key regulatory pathway for the maintenance of appropriate hormone levels to sustain normal growth and survival of prostate epithelial cells. The dysregulation of this pathway is correlated with prostate cancer incidence and progression [22–24]. UGDH is the androgen-stimulated cytosolic enzyme that produces UDP-GlcA [17, 18]. However, the functional role of UGDH in establishing priority use of UDP-GlcA precursors for glucuronidation versus the biosynthetic fates for this metabolite has not been examined in detail. In this study, we provided data in support of significantly altered glucuronidation pathway profiles in human PDX tissues selected for CR recurrence in mice, revealing the potential for coordinated changes in the priority use of UDP-GlcA and the consequences for androgen sensitive outcomes. We further examined the impact of UGDH manipulation in an isogenic AD/CR cell culture model to directly determine the effects of UGDH on gene expression and metabolite levels in the glucuronidation pathway. Overexpression of UGDH reprioritizes the fate of its product, UDP-GlcA, such that AD cells adopt phenotypic characteristics of CR cells. Conversely, UGDH knockdown in CR cells partially restores androgen responsiveness at the molecular level and resensitizes these cells to growth suppression by anti-androgen treatment. Collectively, this is the first study to implicate UGDH as a sensor in the prostate epithelial response to androgens, capable of driving decisions for nucleotide sugar precursor use among multiple fates.

Our first goal in this study was to examine the levels of enzymes, receptors, and metabolites reflecting activity of the glucuronidation pathway relative to the proteoglycan synthesis pathway in PDX tissue. We had previously found several of these components altered in multiple human CR prostate tumor cell lines relative to AD lines, which corresponded to an increase in tumor-promoting proteoglycan production at the expense of androgen glucuronidation, despite apparent increased potential for the latter [17, 18]. In general, the directional changes to multiple components co-associated among CR PDX tissues relative to their counterparts in the same manner that these co-associations occurred in LNCaP during selection of CR cells from the original AD population. Since all five patients had already become castration resistant and had received additional treatments at the time the PDX tissue was collected, it is probable that all original PDX tissues have considerable aberrancies in multiple cellular pathways including those we examined. Some documentation of known oncogenic mutations is summarized in Table 2. For example, all CR PDX had elevated ARv7 relative to CS, which could account for unexpected changes in AR target gene expression due to ligand-independent AR-mediated transcriptional activation. However, it was also reported that intratumoral testosterone and DHT were relatively high in all PDX within the study and that they remained comparable between the CS and the CR PDX tissues. Several points are noteworthy in considering characteristics already reported for these PDX models. Notch1 was elevated in each of the CR PDX models, while UDP-GlcA slightly decreased and UDP-Xyl remained relatively constant in all CR relative to the CS counterpart. This observation, together with unchanged expression of UXS1 and UGDH, is consistent with the interpretation that a common mechanism operates to redirect precursors for androgen elimination into proteoglycan biosynthesis within established tumors as they experience exogenous androgen depletion.

The impact of genetically manipulating UGDH expression in prostate tumor lines has not been previously examined in detail. Particularly since we had found that androgen-stimulated UGDH expression was an important component of androgen homeostasis in AD prostate tumor cells, we expected that overexpression would render the cells hyper-dependent on exogenous androgens for growth and survival by promoting androgen inactivation. Instead, we observed that an elevation in UDP-GlcA selectively supports biosynthetic processes, accelerates cell growth, and opposes the growth suppressive effects of anti-androgen treatment. It is important to emphasize that while the increase in expression of AR splice variants such as ARv7, which can activate AR-mediated gene expression in the absence of ligands, has been shown to be one mechanism by which prostate tumor cells become resistant to anti-androgens and other hormone deprivation therapies (reviewed in [25]), we observed these effects of UGDH overexpression with no detectable increase in ARv7 or other splice variant expression. Therefore, the redirection of nucleotide sugars into biosynthesis by elevated UGDH activity is a novel mechanism for the development of castration resistance and warrants further characterization.

A further significant point about this observation is that previous studies have shown increasing UGDH expression fuels hyaluronan and proteoglycan biosynthesis [26, 27], but none of these studies have been done in the context of a competing priority need for UDP-GlcA in metabolite inactivation. This supports a role for UGDH in functioning as a sensory node for the appropriate channeling of precursors between alternative fates. Because the processes are differentially compartmentalized, with glucuronidation occurring in the ER and most proteoglycans being directed sequentially through layers of Golgi localized enzymatic modification, one intriguing possibility is that UGDH may be recruited to subcellular locations through protein-protein interactions that are conformation dependent. Consistent with a sensing role for UGDH, diminishing UGDH expression by stable knockdown was found to enhance the androgen sensitivity of gene expression and function in an AD background, and to partially restore these features in the CR background (Figure 5). The increased magnitude of response in AR-mediated gene expression exemplified by PSA and UGT2B17 in the absence and presence of androgen, combined with stronger response to enzalutamide, suggests that when UGDH expression is restricted, the limited UDP-GlcA is significantly less available to support biosynthesis of proteoglycans in the Golgi.

There are several possible explanations for the impact of UGDH expression on androgen sensitivity. First, there may be a threshold of UGDH expression and/or activity that promotes increased association between UGDH and the Golgi and/or plasma membrane, where biosynthetic processes are occurring. The association could be favored by reversible post-translational modifications or metabolite binding, as structural studies have revealed the potential existence of alternate conformations for UGDH in the presence and absence of UDP-xylose [28, 29]. Second, a threshold of UDP-sugar availability could dictate incorporation into downstream pathways independently of UGDH conformational recruitment, through differential access to the respective compartments by affinity for nucleotide sugar transporters [30, 31] and/or enzymes utilizing UDP-sugars in those compartments. In fact, we propose that this mechanism would operate in addition to UGDH conformational activation and may be a basal condition that is selectively accelerated by conditions that demand high-level use of UDP-GlcA for a specific output. An increase in flux to proteoglycan synthesis could globally support the expression of cell surface receptors and tumor promoters such as Notch1. If disproportionately augmented, the consequent uncontrolled proliferation and survival may be sufficient to override androgen control of these processes. A third possibility is that excess UDP-sugars may lead to a global increase in glycosylation that skews normal metabolic flux and leads to increased steady state glycosylation of proteins and lipids. An example of this is that UGT2B17 has been reported to undergo glycosylation, but this modification has not yet been associated with a functional implication or a change in stability or subcellular distribution, as might be expected from altered glycosylation. Master transcriptional regulators such as FoxA1 are also glycosylated [32], which can impact their own abundance as well as their target gene expression, so this may partly account for the reduced levels of UGT2B17 and FoxA1 in the LNCaP CR UGDH OE lines (Figure 3A and 3B). Finally, the crosstalk between UDP-sugar metabolism and the hexose biosynthesis pathway may promote altered regulation by O-N-acetylglucosaminylation (O-GlcNAcylation), which is a reversible modification driven by the specific enzyme OGT using UDP-GlcNAc as a precursor (reviewed in [33]). There are numerous metabolic enzymes and epigenetic targets that are impacted by this modification in response to carbohydrate availability. However, this would be a relatively indirect mechanism since we observed no real change in UDP-glucose levels and UDP-glucose is an upstream branch point for hexosamine biosynthesis. Also, to our knowledge, there is no site for O-GlcNAcylation of UGDH or UGT2B17.

When UGDH was overexpressed in either the AD or CR backgrounds, we observed that UGT2B17 levels were elevated and remained high in the presence of DHT. This is consistent with an androgen insensitive, de-repressed state of transcriptional control, and concurs with the other indicators of castration resistant phenotype we monitored. In contrast, UGT2B17 expression was elevated in androgen-depleted conditions when UGDH was inhibited, regardless of the AD or CR background, but suppression of UGT2B17 by androgen was strongly sensitized in both backgrounds relative to the paired UGDH control lines. In addition to requiring FoxA1 for its transcriptional activation, UGT2B17 was recently found to be regulated epigenetically [34]. It is probable that methylation and acetylation are indirectly altered by perturbation of UDP-sugar levels and that this leads to elevated transcription but little change in functional protein. Other than its ER targeting sequence, posttranslational signals or modifications needed to activate UGT2B17 have not been identified. New evidence of alternative promoters [35] and variable splicing [36] suggest the possibility for UGT isoforms to influence activity of glucuronidation in a context dependent manner. Clinically, this is a significant finding, because UGT2B17 elevation correlates with metastatic potential in CRPC [37]. Our model offers an opportunity to investigate mechanisms underlying this association.

The ability to reverse castration resistance even partially by reducing UGDH expression and/or activity is significant. In particular, it would be valuable to determine whether UGDH inhibition combined with anti-androgen treatment at the start of therapy could prevent or delay onset of therapeutic resistance by improving initial response. Understanding the mechanisms underlying the alternate functions of UGDH and how it achieves metabolite partitioning will facilitate effective and more selective targeting.

## MATERIALS AND METHODS

### Reagents and antibodies

Reagents were purchased from Fisher Scientific (Pittsburgh, PA, USA) except as indicated below. 5-α-dihydrotestosterone (DHT) was from Sigma (St. Louis, MO, USA). Charcoal stripped FBS was from Hyclone (Logan, UT, USA). Antibodies were purchased and used as follows: polyclonal rabbit anti-human PSA (DakoCytomation, Glostrup, Denmark, 1:1500 dilution); rabbit polyclonal anti-human MHC class II [EPR11227] (Abcam, Cambridge, MA, USA, 1:1000); mouse monoclonal anti-human β-tubulin (Sigma, 1:750,000); IRDye 800 conjugated anti-rabbit IgG (Rockland, Gilberstville, PA, USA, 1:5000); IRDye 680 conjugated goat anti-mouse IgG (LI-COR Biosciences, Lincoln, NE, USA, 1:5000); rabbit polyclonal anti-human UGT2B17 (GeneTex, Irvine, CA, USA, 1:100), mouse monoclonal anti-human AR (Santa Cruz Biotechnology, Inc, Dallas, TX, USA, 1:500); rabbit monoclonal anti-human Notch1 (Cell Signaling Technology, Danvers, MA, USA, 1:1000); rabbit monoclonal anti-human FoxA1 (Abcam, Cambridge, MA, USA 1:1000). Polyclonal rabbit anti-human UGDH was raised against recombinant UGDH protein purified from *E.coli* conditioned medium and was previously characterized by our laboratory [17]; and polyclonal rabbit anti-human UXS1 was raised against purified recombinant human UXS1 and was previously characterized by our lab [18]. Resazurin cell viability assay kit was from Biotium (Hayward, CA, USA). Enzalutamide was from APExBIO (Houston, TX, USA). Uridine diphosphate-α-D- [1, 2, 3-13C3] glucose (disodium salt) was acquired from Creative Proteomics. Uridine diphosphate-glucose and uridine diphosphate-glucuronate were acquired from MilliporeSigma (St. Louis, MO, USA). Uridine diphosphate-xylose was acquired from the University of Georgia’s CarboSource Services.

### Patient derived xenograft analysis

Frozen samples of five isogenic PDX models from the LuCaP series [19] of CS and CRPC were obtained through the Prostate Cancer Biorepository Network. Tumor specimens were dissected using a sterile razor blade, weighed and then processed as described below for analysis of proteins by immunoblot and quantification of UDP-sugars by liquid chromatography-mass spectrometry (LC-MS).

### Cell culture and selection of stable transfectants

LNCaP (denoted LNCaP AD herein) human prostate adenocarcinoma cell lines were purchased from American Type Culture Collection (Manassas, VA, USA). LNCaP C81 castration resistant cells (denoted LNCaP CR herein) were kindly provided by Dr. Ming-Fong Lin (University of Nebraska Medical Center, Omaha, NE, USA) [21, 38]. For standard culture, LNCaP lines were maintained in RPMI-1640 supplemented with 10% FBS, as recommended by the vendor. To generate UGDH overexpression lines, LNCaP AD and LNCaP CR cells were transduced with lentiviral particles packaged to contain plasmids encoding wild type UGDH with a C-terminal Flag epitope tag, or a vector control expressing EGFP (UGDH EX-Q0483-Lv102 and EX-EGFP-Lv105, respectively, from GeneCopoeia, Rockville, MD, USA). To achieve UGDH knockdown, LNCaP AD and LNCaP CR cells were transfected with plasmids encoding shRNA targeted to UGDH or a scrambled non-targeting control (UGDH shRNA-pGFP-V-RS #TG334012 and 29-mer oligo-pGFP-V-RS #TR30008, respectively, from Origene Technologies, Rockville, MD, USA). Stable integration of the overexpression, shRNA, and respective vector control plasmids was achieved by clonal selection in RPMI-1640 with 10% FBS and 1 μg/ml puromycin. Once selected, clones were maintained in the same medium containing 0.5 μg/ml puromycin. At least six of each group of selected clones were initially characterized for basal and DHT-stimulated responses. Two clones representing the average level of UGDH and PSA expression were chosen from each selected group. UGDH overexpressing (OE) lines were defined as those with >50% overexpressed UGDH protein relative to the vector control (VC) clones, in which UGDH expression was identical to the parental line. UGDH knockdown (KD) clones were defined by >70% reduced UGDH protein expression relative to UGDH expression in VC clones that were identical to the parental line. UGDH overexpression or knockdown was confirmed by western analysis of whole cell lysates, probed first with anti-FLAG, then stripped and re-probed with anti-UGDH. Vector control clones were referred to as VC1 and VC2, UGDH overexpression clones were designated OE1 and OE2, and UGDH knockdown clones were called KD1 or KD2. All cell lines used in this study were authenticated by ATCC using STR profiling.

### Androgen stimulation

For short term comparison of androgen response, LNCaP and clonally derived lines were subcultured to 50% confluence in phenol red-free RPMI-1640 supplemented with 1% charcoal stripped FBS (CS-FBS) for 48 h (androgen free conditions). DHT was serially diluted into media from concentrated stocks. Growth media were replaced with 2 ml per well of media containing the indicated concentration of DHT for 48 h, and harvested for analysis.

### Western analysis

Cells were washed 1x with PBS, scraped into cold PBS following treatments, pelleted by centrifugation, and lysed in 1x RIPA buffer with protease inhibitor cocktail. Protein was quantified by Bradford assay and equivalent amounts of total protein were analyzed. PDX samples were homogenized in 1x RIPA buffer containing protease inhibitor cocktail (1 mL per 100 mg of tissue) and centrifuged to clear debris. Protein was quantified by Pierce bicinchoninic acid assay (Thermo Scientific) and equivalent amounts of total protein were analyzed. UGDH, PSA, AR, and β-tubulin were probed simultaneously in cell lysates. UXS1 and β-tubulin were co-detected and blots were stripped and re-probed for UGT2B17 and β-tubulin. Notch1, FoxA1, and β-tubulin, were detected simultaneously. Human MHCII and β-tubulin were detected simultaneously. Following secondary incubation, protein expression was quantified by fluorescence emission using the Odyssey Infrared Imaging System (LI-COR Biosciences, Lincoln, NE, USA). Signals for each wavelength were analyzed in red (700 nm, AR and β-tubulin) and green (800 nm, all other targets), to normalize protein expression. Each densitometric analysis was plotted for three technical replicates of a representative experiment that was repeated at least three times. Statistical significance was assessed by one-way ANOVA followed by Tukey’s post hoc test for multiple comparisons.

### Quantification of UDP-sugars

Lysates from the indicated lines and treatments were extracted with butanol-saturated concentrated formic acid and analyzed for UDP-sugar content by LC-MS according to published methods [39, 40], with minor modifications described below. Butanol based Uridine diphosphate-α-D- [1, 2, 3-13C3] glucose was added as an internal standard solution. UDP-Glc, UDP-GlcA, and UDP-Xyl analytical standards were used for quantitative purposes to produce a calibration curve that was run daily and ranged from 200 ng/mL to 10,000 ng/mL. All LC-MS analyses were performed on a TSQ Altis triple quadrupole instrument (Thermo Scientific, San Jose, CA, USA), operated in negative ionization mode, incorporating a Thermo Scientific Vanquish LC system (Germering, Germany) with a Thermo Hypercarb Porous Graphitic Carbon column (100 mm × 2.1 mm, 3 μm particle size). Solvents A (water with 0.6% ammonium hydroxide and 0.1% formic acid, pH 9.1) and B (acetonitrile with 5% water and 0.1% formic acid) were used to provide a 0.25 mL/min gradient as follows: (time/%B): Initial/5%, 0.25 min/5%, 5 min/95%, 7.5 min/95%, 7.52 min/5%, 10 min/5%. The mass spectrometer was run using a capillary voltage of (-)2500V, a sheath gas of 50 (arbitrary units), an aux gas of 10 (arbitrary units), a sweep gas of 1 (arbitrary units), an ion transfer tube temperature of 325°C, and a vaporizer temperature of 350°C. Transition data were integrated and tabulated using Skyline software (version 20.2.1.454) developed by the MacCoss Lab at University of Washington [41]. Four technical replicates were analyzed in each experiment, repeated three times. Statistical significance was assessed by one-way ANOVA followed by Tukey’s post hoc test for multiple comparisons.

### Cell proliferation assay

For basal growth, LNCaP AD lines (2 × 10^4^ cells) and LNCaP CR lines (1 × 10^4^ cells) selected for UGDH overexpression, UGDH knockdown, or vector control expression, were cultured in 24-well plates in standard steroid-replete media. Cell proliferation was measured daily in replicate plates incubated for 2 h (LNCaP AD) or 3 h (LNCaP CR) with media containing 10% resazurin, after which fluorescence of the reduced product, resorufin, was measured (560_Ex_/590_Em_) in a microplate fluorometer. For androgen-mediated cell growth, LNCaP AD (2 × 10^4^) and LNCaP CR (1 × 10^4^) sublines were cultured in 24-well plates in standard steroid-replete media. After 24 h, media were first removed and replaced with media containing 5% CS-FBS (androgen-depleted conditions) for 48 h, and then media were removed and replaced with media containing 5% CS-FBS plus vehicle (0 nM) or 10 nM DHT for another 48 h. Cell proliferation was measured daily as described above beginning when cells were switched to androgen free conditions. For enzalutamide treatments, LNCaP AD (2 × 10^4^) and LNCaP CR (1 × 10^4^) sublines were cultured in 24-well plates in standard steroid-replete media. After 24 h, replete media containing vehicle (0 μM) or enzalutamide were added in equal volume to the wells for final treatment of 0 μM, 0.1 μM, 1 μM, and 10 μM for another 72 h. Cell proliferation was measured daily as described above beginning when cells were switched to enzalutamide treatment. All data were plotted for four technical replicates of experiments that were repeated three times. Statistical significance was assessed by one-way ANOVA followed by Tukey’s post hoc test for multiple comparisons.

## SUPPLEMENTARY MATERIALS



## Abbreviations

AD: androgen dependent
CS: castration sensitive
CR: castration resistant
PDX: patient-derived xenograft
AR: androgen receptor
UGDH: UDP-glucose dehydrogenase
UGT: UDP-glucuronosyltransferase
UDP-Glc: UDP-Glucose
UDP-GlcA: UDP-Glucuronate
UDP-Xyl: UDP-Xylose
OE: overexpression
KD: knockdown
VC: vector control
PSA: prostate specific antigen
DHT: dihydrotestosterone
