# Supplementary material for: Altered glucuronidation deregulates androgen dependent response profiles and signifies castration resistance in prostate cancer
Source: Oncotarget. 2021 Sep 14;12(19):1886–902. doi: 10.18632/oncotarget.28059 (PMC8448517; doi:10.18632/oncotarget.28059)
Supplement: Supplementary file 1 [file oncotarget-12-1886-s001.pdf]

Altered glucuronidation deregulates androgen dependent response profiles and signifies castration resistance in prostate cancer

SUPPLEMENTARY MATERIALS

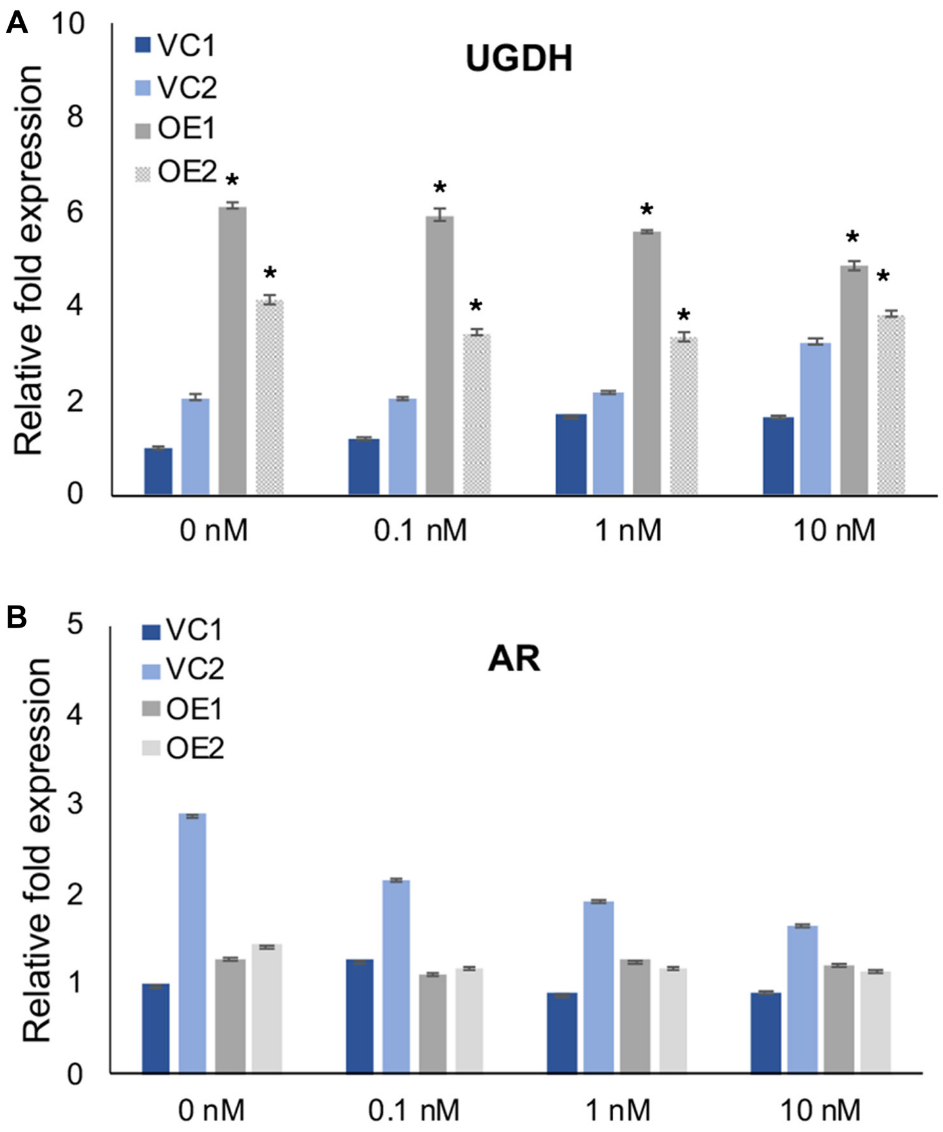

Supplementary Figure 1: UGDH and AR expression in LNCaP AD lines VC1, VC2, OE1 and OE2. Cells were incubated in the presence and absence of DHT as indicated (in nM), and assayed by western blot, followed by densitometric analysis relative to tubulin. Mean ± SEM is plotted. $^{*}p < 0.05$ relative to VC1 or VC2 at all [DHT]. (**A**) Relative UGDH expression. (**B**) Relative AR expression.

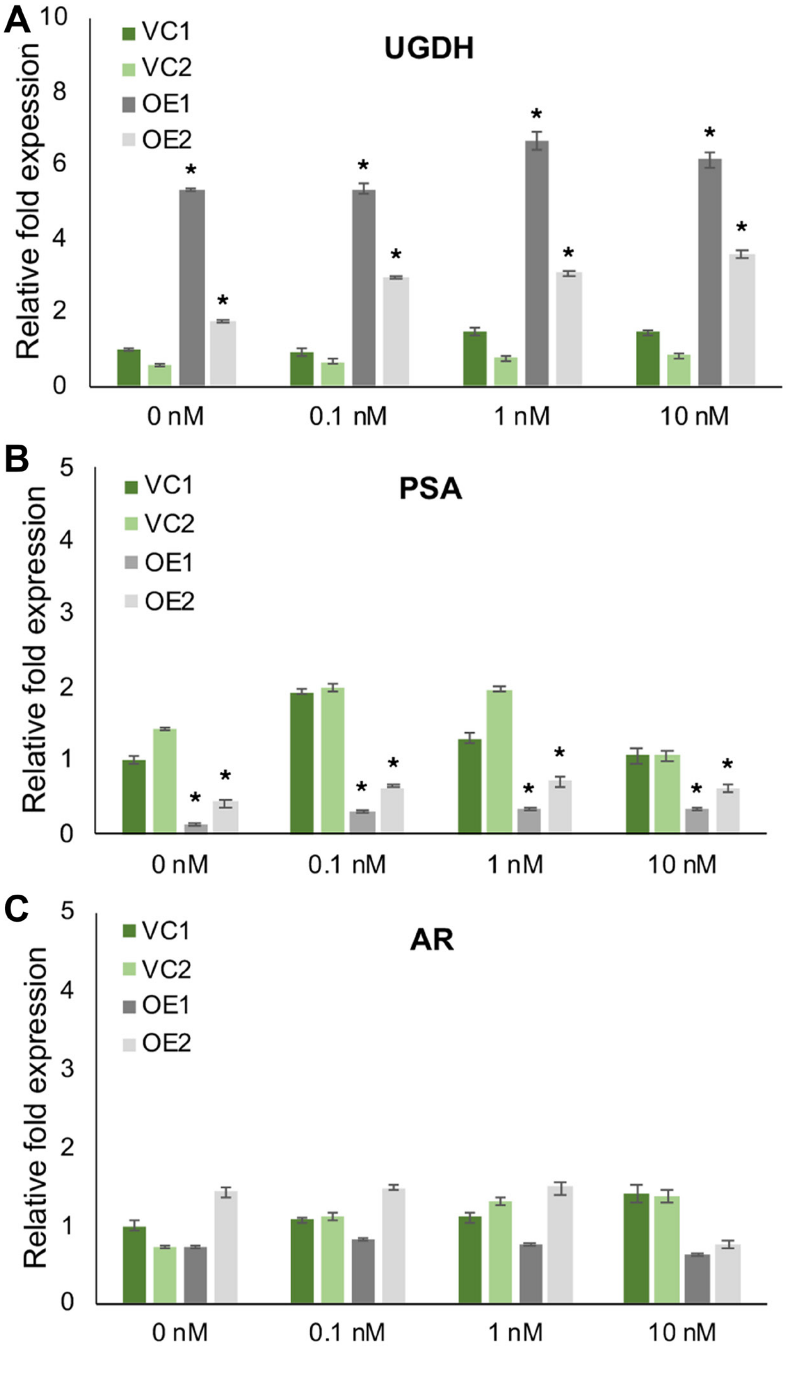

Supplementary Figure 2: UGDH, PSA, and AR expression in LNCaP CR lines VC1, VC2, OE1 and OE2. Cells were incubated in the presence and absence of DHT as indicated (in nM), and assayed by western blot, followed by densitometric analysis relative to tubulin. Mean ± SEM is plotted. $^{*}p < 0.05$ relative to VC1 or VC2 at all [DHT]. (**A**) Relative UGDH expression. (**B**) Relative PSA expression. (**C**) Relative AR expression.

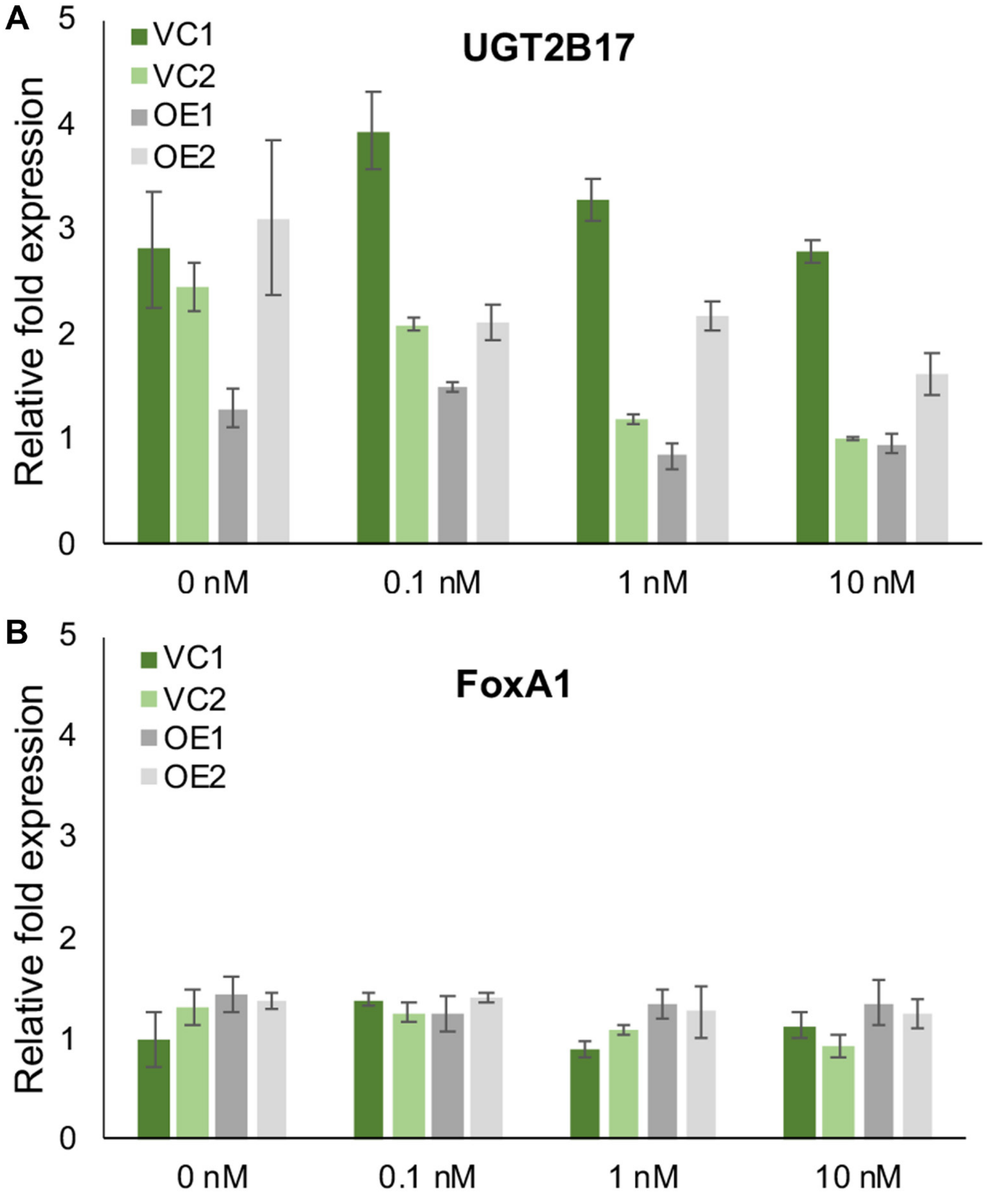

Supplementary Figure 3: UGT2B17 and FoxA1 expression in LNCaP CR lines VC1, VC2, OE1, OE2 in the presence and absence of DHT (in nM) assayed by western blot and densitometric analysis. Mean ± SEM is plotted for technical triplicates of a representative assay. No consistent, statistically significant differences were found between three replicate assays. (**A**) Relative UGT2B17 expression. (**B**) Relative FoxA1 expression.

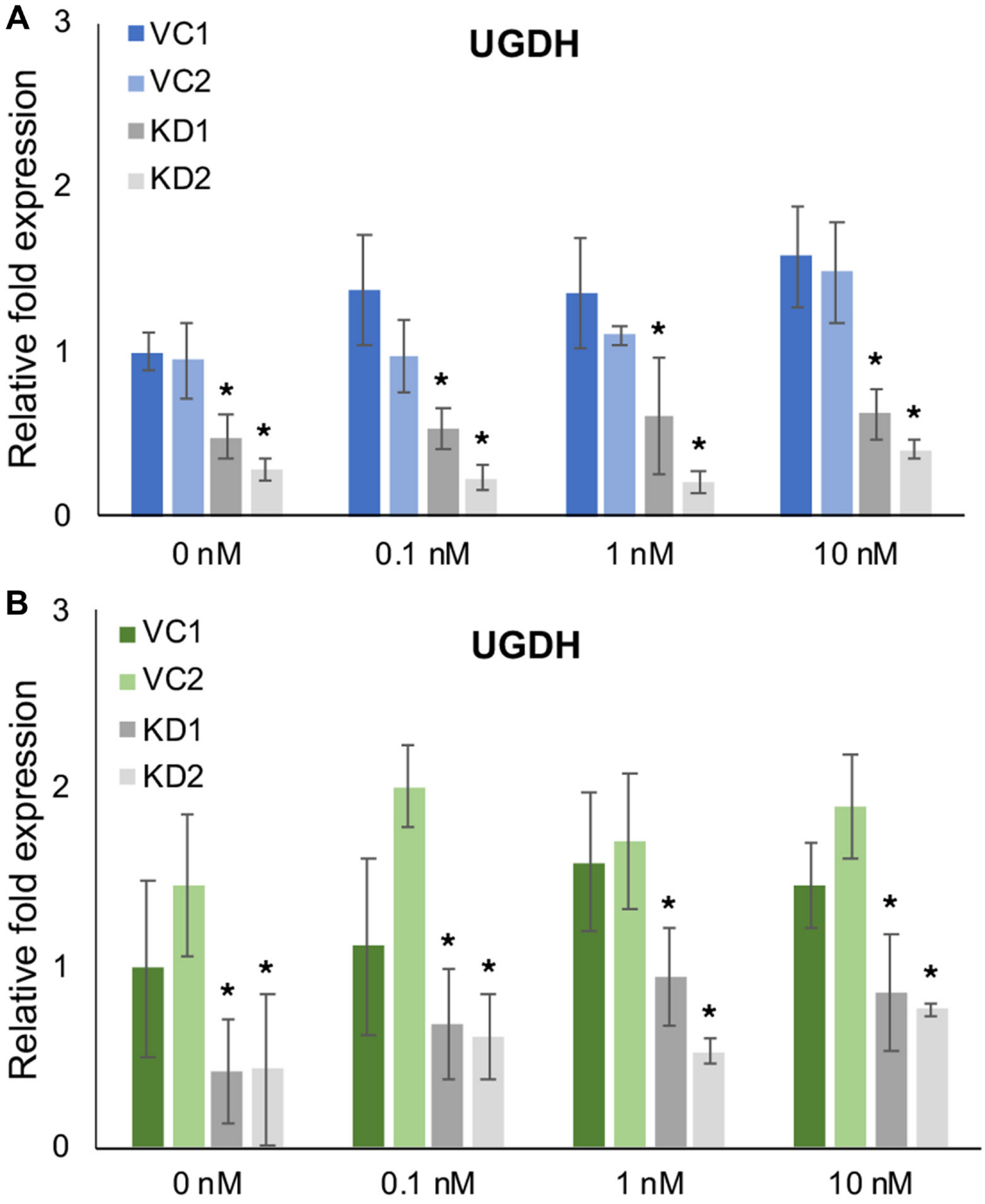

Supplementary Figure 4: UGDH expression in LNCaP knockdown cells. Cells were incubated in the presence and absence of DHT as indicated (in nM), and assayed by western blot, followed by densitometric analysis relative to tubulin. Mean ± SEM is plotted. $^{*}p < 0.05$ relative to VC1 or VC2 at all [DHT]. (**A**) LNCaP AD clones VC1, VC2, KD1 and KD2. (**B**) LNCaP CR clones VC1, VC2, KD1 and KD2.

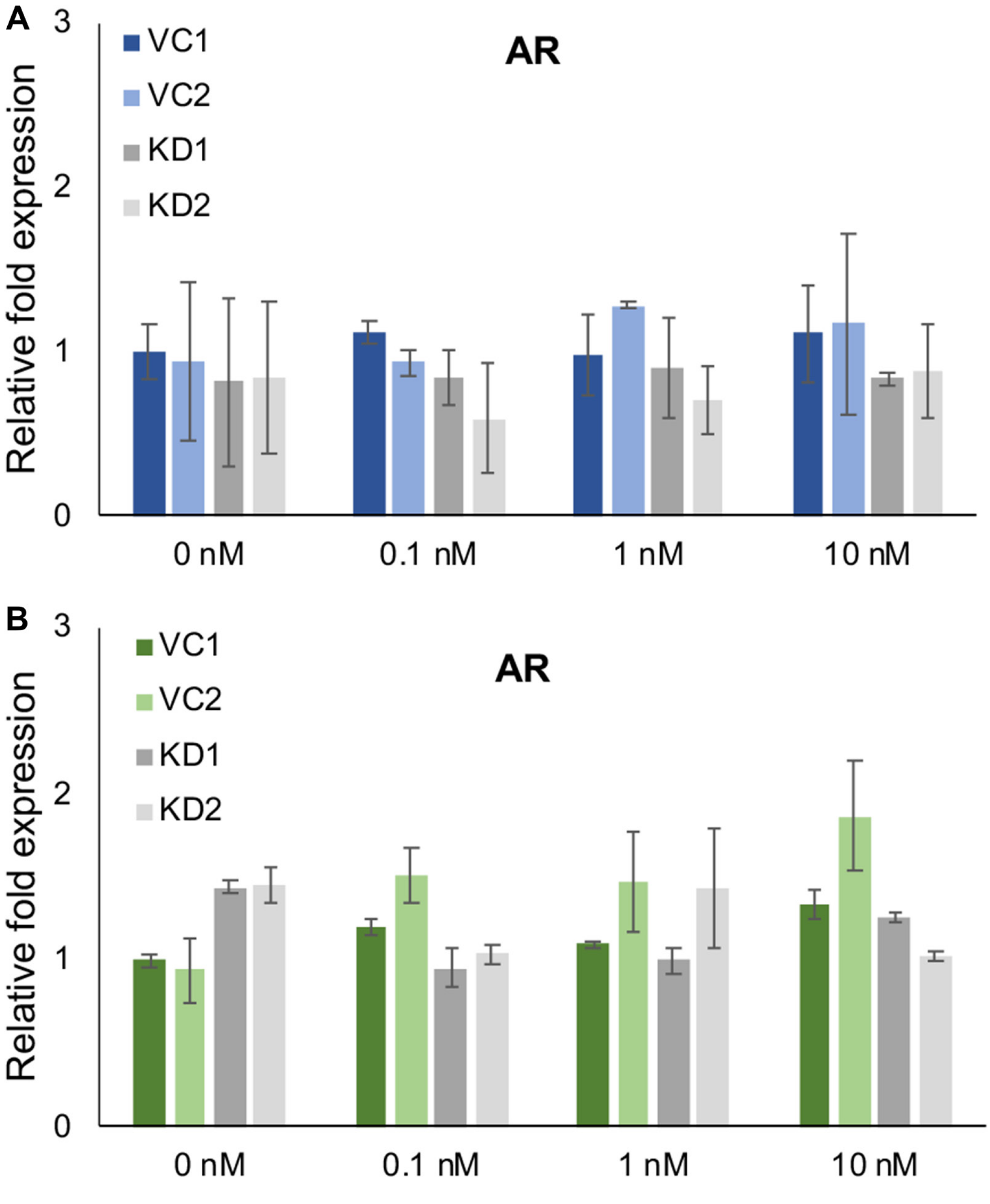

Supplementary Figure 5: AR expression in the presence and absence of DHT (in nM). Cell lysates were assayed by western blot and densitometric analysis. Mean ± SEM is plotted for technical triplicates of a representative assay. No consistent, statistically significant differences were found between three replicate assays. (**A**) LNCaP AD clones VC1, VC2, KD1 and KD2. (**B**) LNCaP CR clones VC1, VC2, KD1 and KD2.

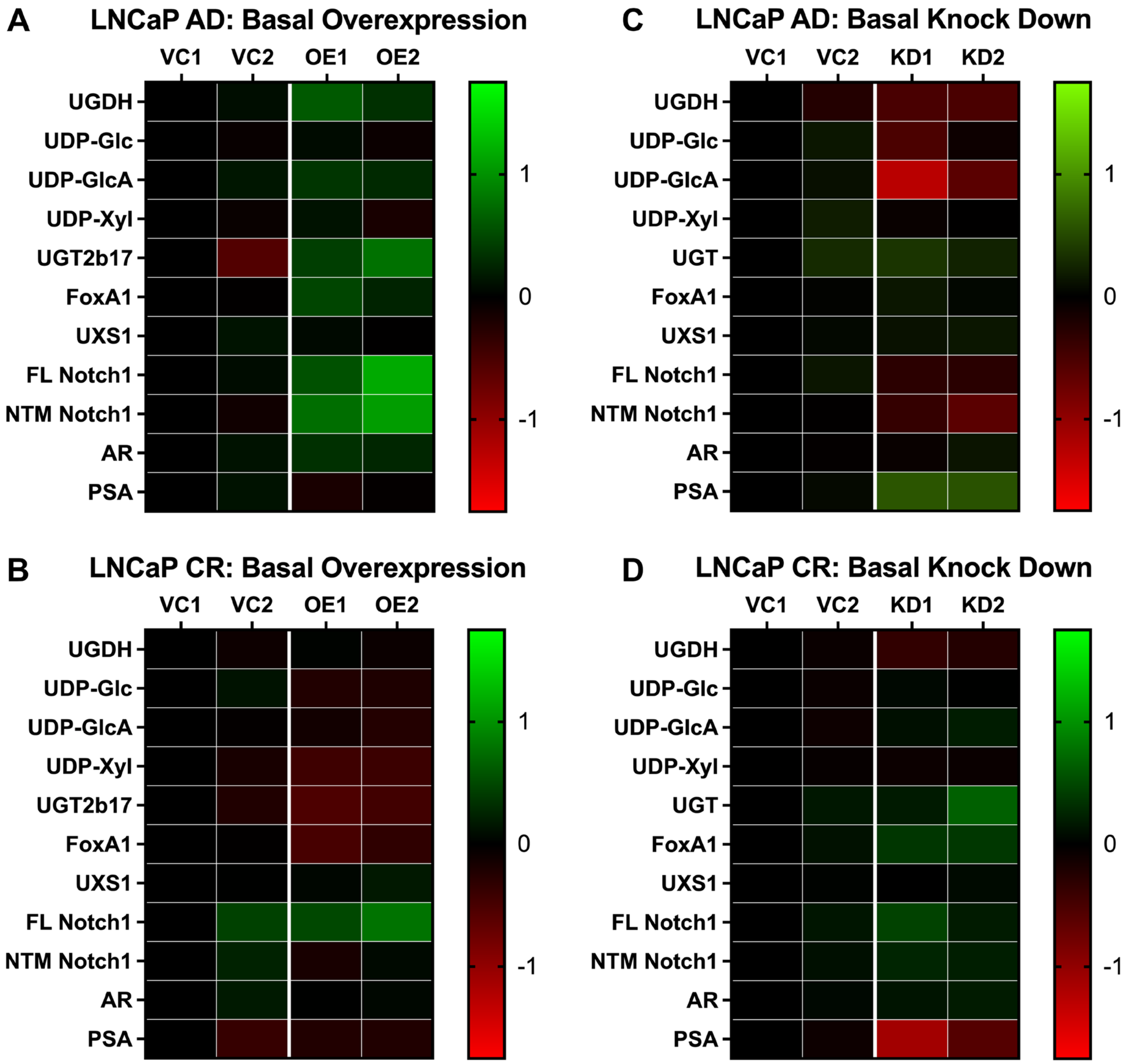

Supplementary Figure 6: Heat map summaries of gene expression and metabolite levels in androgen replete (standard media) conditions for all LNCaP stable lines in this study. (**A**) LNCaP AD: VC1, VC2, OE1 and OE2. (**B**) LNCaP CR: VC1, VC2, OE1 and OE2. (**C**) LNCaP AD: VC1, VC2, KD1 and KD2. (**D**) LNCaP CR: VC1, VC2, KD1 and KD.

Supplementary Table 1: Summary of PDX analysis data

	UGDH		AR		PSA		UGT2B17		FoxA1	
**PDX**	AVG	SEM	AVG	SEM	AVG	SEM	AVG	SEM	AVG	SEM
35	6081.797	2621.387	0.207439	0.121739	153.8987	80.03333	6.082016	5.786448	43.72533	7.672095
35CR	3372.67	2818.711	43.72428	42.93949	730.0113	359.7509	8.82907	5.457579	408.8726	218.1072
70	5889.486	2527.798	0.218073	0.077746	1926.155	931.022	5.436722	4.950628	13.44789	6.456604
70CR	967.0483	361.019	3.791157	3.238912	192.5884	100.7285	1.034572	0.467358	189.5833	138.8172
73	3531.301	986.1269	0.626632	0.288289	3858.428	2011.609	9.111407	8.551982	431.165	294.0716
73CR	527.1651	243.5313	1.507589	1.180627	326.125	108.8948	3.69946	3.388315	1101.582	834.9526
77	700.2079	450.1768	1.406759	0.796961	2363.417	1832.864	5.415568	5.349139	1243.99	1025.934
77CR	2395.406	320.9297	0.34817	0.231686	1552.423	476.5753	5.725311	5.057324	14.6924	6.389439
78	1758.75	1386.234	0.534968	0.530682	8329.478	6985.702	13.95689	13.3537	2266.317	2137.625
78CR	447.4017	199.5643	0.266581	0.115768	2373.786	1263.239	19.25534	18.9938	512.9025	343.0869

	UXS1		FL Notch1		NTM Notch1		UDP-Glc		UDP-GlcA		UDP-Xyl	
**PDX**	AVG	SEM	AVG	SEM	AVG	SEM	AVG	SEM	AVG	SEM	AVG	SEM
35	25.56128	12.35877	0.114336	0.052877	10.50659	4.710941	28.69659	0.741416	6.856845	0.123088	3.13796	0.065318
35CR	67.2848	34.98683	7.460705	4.745312	81.89918	31.58057	7.449272	0.148585	3.004916	0.076579	1.466038	0.028149
70	34.17581	27.85825	0.106423	0.043996	2.22048	1.073715	22.00297	0.601857	4.154504	0.122034	2.051604	0.051982
70CR	4.637109	1.499665	0.88033	0.281072	30.67324	12.99841	30.56777	1.037419	3.752115	0.097089	2.29086	0.100855
73	62.71423	31.02339	0.880982	0.687656	54.98429	52.13365	15.93269	0.068953	12.04567	0.085619	0.847021	0.008717
73CR	37.71919	15.21936	13.37932	7.910484	53.22274	22.11425	27.94636	0.423033	4.982188	0.155922	1.104568	0.031804
77	25.63538	18.69072	0.182929	0.118707	15.6387	10.89852	32.6933	1.379913	5.841235	0.240743	1.695015	0.077248
77CR	33.68304	9.377558	0.410634	0.130368	2.214967	0.381835	2.704074	0.102177	1.058792	0.044252	0.290625	0.004113
78	111.1579	63.39076	5.468828	2.439215	256.4028	244.7156	6.283618	0.134129	8.827111	0.143124	1.208538	0.032426
78CR	52.04574	41.96551	2.697154	0.461806	39.06587	26.17153	7.107744	0.240452	5.325954	0.155484	1.689428	0.097622

*Gene expression was analyzed by western blot; all values are normalized to MHCII, probed simultaneously and quantified ratiometrically as detailed in Materials and Methods. UDP-sugar data were measured by LC-MS and are given in nmol/mg of protein.

Supplementary Table 2: Summary of LNCaP subclone analysis data*

	UGDH		AR		PSA		UGT2B217		FoxA1	
	AVG	SEM	AVG	SEM	AVG	SEM	AVG	SEM	AVG	SEM
**LNCaP AD**	2.639159	0.093811	1.211163	0.301172	2.71206	0.297906	0.463009	0.043556	1.684355	0.270172
UGDH VC1	0.1618	0.030335	0.020413	0.003973	0.3261	0.031379	0.052327	0.009805	0.167696	0.045825
UGDH VC2	0.1107	0.033585	0.017254	0.000978	0.3701	0.075871	0.043908	0.005444	0.173435	0.075635
UGDH KD1	0.0895	0.03491	0.117249	0.023577	0.5326	0.063826	0.065039	0.012067	0.129248	0.020555
UGDH KD2	0.0753	0.014716	0.091314	0.005772	1.2474	0.125203	0.09122	0.001649	0.18392	0.008776
UGDH EV1	1.754242	0.385099	0.937012	0.163578	2.765219	0.281583	0.376042	0.0722	1.814863	0.206805
UGDH EV2	2.196553	0.71792	0.735501	0.085682	2.551565	0.112547	0.480431	0.083753	1.659163	0.223477
UGDH OE1	6.686065	0.600624	0.652025	0.107583	0.559889	0.079847	1.37744	0.274005	4.914208	0.91662
UGDH OE2	10.55504	1.885689	0.687214	0.053339	0.90499	0.013532	3.512035	0.549901	2.861257	0.211021

	UXS1		FL Notch1		NTM Notch1		UDP-Glc		UDP-GlcA		UDP-xylose	
	AVG	SEM	AVG	SEM	AVG	SEM	AVG	SEM	AVG	SEM	AVG	SEM
**LNCaP AD**	0.051151	0.002085	0.006993	0.001591	0.289618	0.073277	1.816435	0.79788	0.607596	0.11121	0.431415	0.08373
UGDH VC1	0.0126	0.001578	0.121524	0.003	0.023741	0.007586	0.17291	0.00609	0.222663	0.01184	0.018313	0.00115
UGDH VC2	0.0140	0.002225	0.087794	0.014962	0.022937	0.003758	0.104827	0.00566	0.281669	0.05654	0.012862	0.00106
UGDH KD1	0.0165	0.001923	0.043806	0.023027	0.010416	0.003105	0.140134	0.00308	0.0071	3.00E-04	0.0044	7.65E-05
UGDH KD2	0.0177	0.001774	0.045421	0.014	0.005669	0.001751	0.139963	0.00934	0.053361	0.01251	0.007992	0.00041
UGDH EV1	0.056964	0.008977	0.007817	0.001711	0.249875	0.035629	0.88179	0.28842	0.797408	0.25734	0.249548	0.09899
UGDH EV2	0.048378	0.001448	0.010605	0.005397	0.454422	0.119184	0.773878	0.25997	0.721379	0.15232	0.213239	0.08189
UGDH OE1	0.065071	0.006081	0.025735	0.002371	0.839054	0.135892	0.859168	0.23240	1.164992	0.19600	0.222848	0.04283
UGDH OE2	0.055734	0.004935	0.018974	0.000788	0.949407	0.097704	0.778217	0.27721	1.093979	0.28299	0.174151	0.05038

*Gene expression was analyzed by western blot; all values are normalized to Tubulin, probed simultaneously and quantified ratiometrically as detailed in Materials and Methods. UDP-sugar data are given in nmol/million cells.

	UGDH		AR		PSA		UGT2B17		FoxA1	
	AVG	SEM	AVG	SEM	AVG	SEM	AVG	SEM	AVG	SEM
**LNCaP CR**	4.147754	0.360326	1.441548	0.166739	0.492456	0.097911	1.177323	0.186879	6.50063	1.109056
UGDH VC1	5.397728	0.067438	1.102965	0.018812	2.09105	0.133042	0.227314	0.008332	1.782469	0.446101
UGDH VC2	4.705717	0.082776	1.239333	0.016831	1.702492	0.241893	0.324374	0.031764	2.301977	0.31621
UGDH KD1	3.056545	0.134114	1.502634	0.079326	0.159318	0.014552	0.339332	0.043178	4.137812	0.185045
UGDH KD2	3.072138	0.172032	1.68103	0.154068	0.553523	0.055624	1.030477	0.025634	4.211831	0.764841
UGDH EV1	4.316107	0.118493	1.005196	0.096437	0.301663	0.025201	1.185996	0.466379	2.618253	0.430241
UGDH EV2	3.919878	0.051935	1.140746	0.079768	0.369051	0.013052	1.914567	0.171767	2.528472	0.220311
UGDH OE1	7.736747	0.180577	1.366543	0.096792	0.239678	0.019003	0.36522	0.045792	0.847919	0.208304
UGDH OE2	6.311697	0.364574	1.298963	0.218346	0.198729	0.027603	0.35267	0.081224	1.238831	0.058807

	UXS1		FL Notch1		NTM Notch1		UDP-Glc		UDP-GlcA		UDP-xylose	
	AVG	SEM	AVG	SEM	AVG	SEM	AVG	SEM	AVG	SEM	AVG	SEM
**LNCaP CR**	0.025731	0.005729	0.042134	0.004902	1.084005	0.339868	0.797716	0.322151	1.550292	0.287243	0.170168	0.01059
UGDH VC1	0.033045	0.001592	0.023435	0.005671	1.004938	0.063559	30.34756	2.953063	75.85319	9.099002	0.87294	0.07774
UGDH VC2	0.034626	0.006043	0.029007	0.002229	1.122215	0.035892	25.77202	3.524726	61.47739	8.894368	0.78231	0.08708
UGDH KD1	0.03288	7.69E-05	0.091929	0.005877	1.166316	0.036094	33.99147	9.725733	95.08412	17.551	0.718452	0.13663
UGDH KD2	0.038504	0.004125	0.02511	0.005755	1.051731	0.105455	30.66253	6.242124	117.9478	13.53122	0.735662	0.10927
UGDH EV1	0.025507	0.00116	0.003681	0.00179	0.222118	0.032025	0.458422	0.26467	0.601456	0.347251	0.31927	0.18433
UGDH EV2	0.025803	0.000939	0.010273	0.002174	0.378577	0.046869	0.953112	0.55028	0.333528	0.192563	0.083546	0.04823
UGDH OE1	0.027697	0.001276	0.011391	0.002027	0.150885	0.016595	0.28297	0.163373	0.095542	0.055161	0.062984	0.03636
UGDH OE2	0.037246	0.001599	0.022319	0.000978	0.253895	0.015639	0.329721	0.190364	0.097923	0.056536	0.057792	0.03336

*Gene expression was analyzed by western blot; all values are normalized to Tubulin, probed simultaneously and quantified ratiometrically as detailed in Materials and Methods. UDP-sugar data are given in nmol/million cells.
